# Optimizing cancer treatment: the synergistic potential of CAR-T cell therapy and CRISPR/Cas9

**DOI:** 10.3389/fimmu.2024.1462697

**Published:** 2024-11-08

**Authors:** Maryam Amiri, Amir Kian Moaveni, Masoumeh Majidi Zolbin, Behrouz Shademan, Alireza Nourazarian

**Affiliations:** ^1^ Pediatric Urology and Regenerative Medicine Research Center, Children’s Medical Center, Tehran University of Medical Sciences, Tehran, Iran; ^2^ Medical Journalism, School of Paramedical Sciences, Shiraz University of Medical Sciences, Shiraz, Iran; ^3^ Department of Basic Medical Sciences, Khoy University of Medical Sciences, Khoy, Iran

**Keywords:** CAR-T cell therapy, CRISPR/Cas9 technology, immunotherapy, genetic engineering, personalized cancer treatment

## Abstract

Optimizing cancer treatment has become a pivotal goal in modern oncology, with advancements in immunotherapy and genetic engineering offering promising avenues. CAR-T cell therapy, a revolutionary approach that harnesses the body’s own immune cells to target and destroy cancer cells, has shown remarkable success, particularly in treating acute lymphoblastic leukemia (ALL), and in treating other hematologic malignancies. While CAR-T cell therapy has shown promise, challenges such as high cost and manufacturing complexity remain. However, its efficacy in solid tumors remains limited. The integration of CRISPR/Cas9 technology, a powerful and precise genome-editing tool, also raises safety concerns regarding unintended edits and off-target effects, offers a synergistic potential to overcome these limitations. CRISPR/Cas9 can enhance CAR-T cell therapy by improving the specificity and persistence of CAR-T cells, reducing off-target effects, and engineering resistance to tumor-induced immunosuppression. This combination can also facilitate the knockout of immune checkpoint inhibitors, boosting the anti-tumor activity of CAR-T cells. Recent studies have demonstrated that CRISPR/Cas9-edited CAR-T cells can target previously untreatable cancer types, offering new hope for patients with refractory cancers. This synergistic approach not only enhances the efficacy of cancer treatment but also paves the way for personalized therapies tailored to individual genetic profiles. This review highlights the ongoing research efforts to refine this approach and explores its potential to revolutionize cancer treatment across a broader range of malignancies. As research progresses, the integration of CAR-T cell therapy and CRISPR/Cas9 holds the promise of transforming cancer treatment, making it more effective and accessible. This review explores the current advancements, challenges, and future prospects of this innovative therapeutic strategy.

## 1 Introduction

Cancer continues to be a major health challenge in modern times, with its burden increasing globally. The complexity of this disease and its high levels of genetic diversity have prompted scientists to consider more tailored approaches to treatment. Typical approaches in cancer treatment include surgery, radiation, and systemic drugs, which are usually used alone or in combination. Cytotoxic therapies for most people bring about significant suffering and do not provide long-term immunity against the disease (1).

Alongside the growing demand for cancer treatment, the cost per patient has consistently risen. Consequently, healthcare spending on cancer care has increased at a rate that surpasses the rise in cancer incidence (2). The pursuit of optimizing cancer treatment stands at the forefront of modern oncology, driven by the critical need to enhance patient outcomes and overcome the limitations of existing therapies. In response, advancements in immunotherapy and genetic engineering have emerged as promising avenues, offering more targeted and effective solutions.

What has been envisioned over the past decade is that immunotherapy has quickly transformed from a fancy concept to a practical and revolutionary method for treating cancer. One of the most promising and innovative approaches among these strategies is chimeric antigen receptor T (CAR-T) cell therapy. This marks a significant milestone in cancer treatment, departing from traditional methods that aimed to harness the body’s immune system. More specifically, CAR T-cell therapy has shown tremendous potential for targeting and combating cancer cells in ways previously unattainable through conventional treatment methods (3). To achieve this, the patient’s immune cells are genetically engineered to express chimeric receptors that specifically bind to antigens and activate cytotoxic T lymphocytes. This enhancement boosts the immune cells’ efficiency in targeting and destroying cancer cells (4). In 2008, Malcolm Brenner and colleagues in Houston reached a pivotal achievement in the clinical application of CAR T cells (5). This method has demonstrated notable efficacy in managing blood cancers such as leukemia and lymphoma, resulting in substantial remission rates for patients with few prior treatment options (6). Although the potential of CAR T-cell therapy is promising, it still poses several challenges that researchers must overcome. The treatment has been noted for its success, although some patients are subject to severe side effects, toxicity of treatment, or inefficacy, in which case they face a recurrence of cancer (7–9). Scientists are thus probing different strategies to tackle these, such as optimization of the CAR structure, combination therapies with radiotherapy and chemotherapy, immune checkpoint inhibitors, and oncolytic viruses (10–13). In this regard, although these strategies have achieved improvement with respect to their efficacy and safety, they have not been able to completely alleviate all the concerns. Besides, such high treatment costs have also prevented the large-scale clinical application of CAR-T cell therapy. So, there is a continued need to further refine this technology for better effectiveness and safety, together with a lower manufacturing cost (14). These efforts would further make CAR-T cell therapy more feasible and accessible to a larger population of cancer patients.

The clustered regularly interspaced short palindromic repeat (CRISPR)/Cas9 technology is a widely recognized genome editing tool that utilizes guide RNA (sgRNA) to target specific DNA sequences. It has garnered significant interest due to its ability to target multiple genes simultaneously, its ease of implementation, and its cost-effectiveness (15). A significant breakthrough was made by Zhang Feng and et al. (16) to demonstrate the effectiveness of CRISPR/Cas9 technology in human and mammalian cells. This advancement has expanded the capabilities of CAR-T cell therapy. Currently, researchers are utilizing the CRISPR/Cas9 system to edit and engineer CAR-T cells, enhancing their ability to target cancer. Specifically, these scientists are modifying the cells to improve specificity, target a greater range of antigens, prolong persistence, broaden the scope of action against cancer, and enhance safety. Of particular interest is the application of CRISPR/Cas9 in engineering CAR-T cells (17). This powerful gene-editing tool allows for precise genetic changes, thereby improving the specificity and efficacy of CAR-T cells. Furthermore, it enables the development of CAR-T cells that can overcome immunosuppression in the tumor microenvironment and potentially target a wider array of cancer types. CRISPR/Cas9 technologies will unlock these possibilities and more, presenting a flexible and highly promising option for CAR-T cell therapy for most cancer patients, thus mitigating current limitations. This review explores the synergistic potential of combining CAR-T cell therapy with CRISPR/Cas9 technology. By delving into the latest advancements, current challenges, and future prospects, we aim to shed light on how this innovative therapeutic strategy can transform cancer treatment, making it more effective, accessible, and personalized for patients worldwide.

## 2 CAR-T cell therapy

CAR-T cell therapy is a revolutionary approach in cancer treatment that leverages genetically engineered T cells to target and eliminate cancer cells. By introducing chimeric antigen receptors (CARs) into T cells, CAR-T therapy equips them with the ability to recognize and destroy specific cancer cells. CARs are artificial proteins composed of an extracellular antigen-binding domain, a hinge region, a transmembrane domain, and an intracellular signaling domain. The antigen-binding domain, typically derived from a single-chain variable fragment (scFv) of a monoclonal antibody, recognizes and binds to specific antigens on the surface of cancer cells. Upon binding to the target antigen, the CAR triggers a cascade of signaling pathways within the T cell, leading to its activation, proliferation, and release of cytotoxic molecules that destroy the cancer cell (6). However, CAR-T cell therapy also faces challenges, such as potential for severe side effects, high manufacturing costs, and limited efficacy in solid tumors.

### 2.1 Mechanism of action

**Figure 1:**
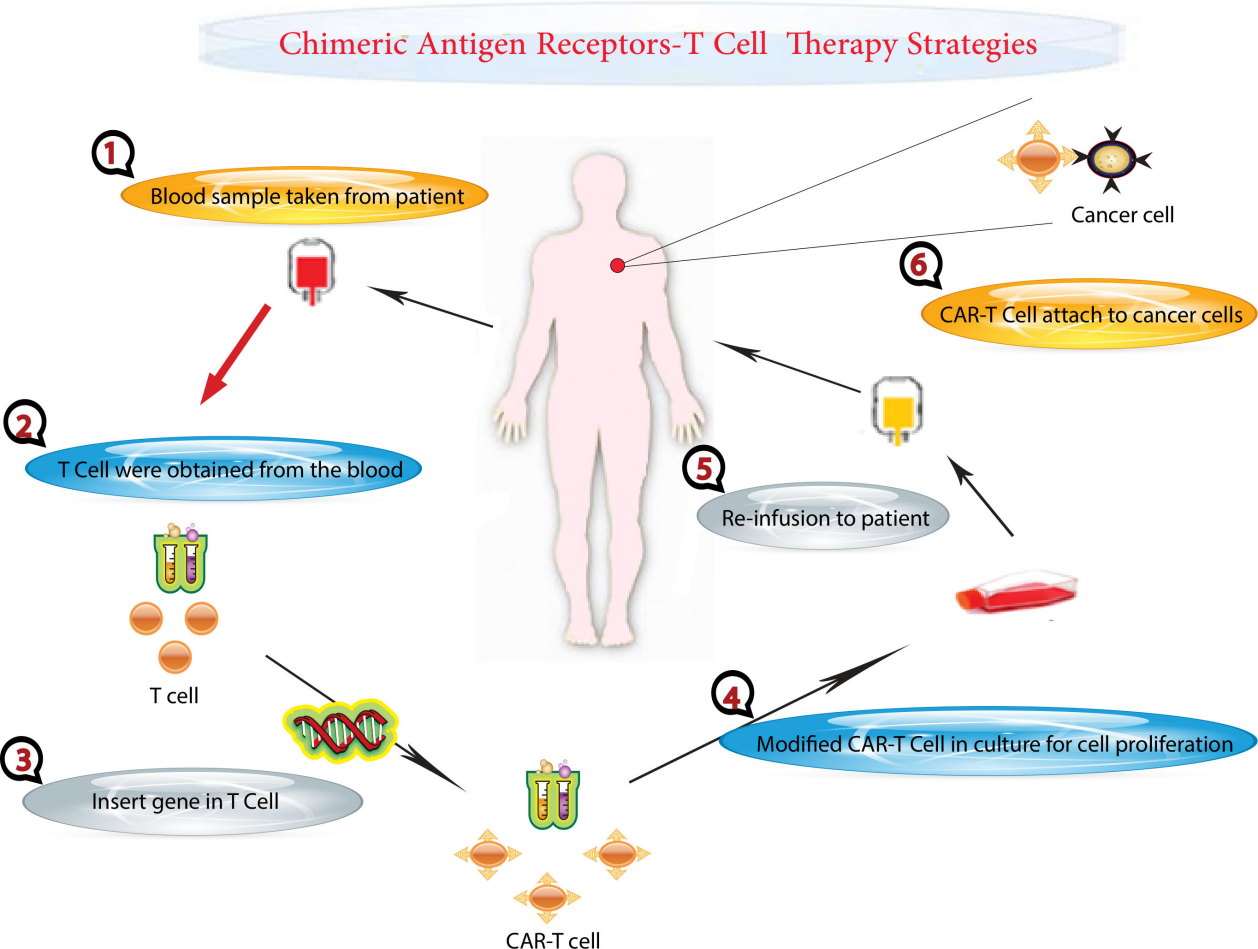
CAR T-Cell Therapy Process. This figure illustrates the step-by-step process of CAR T-cell therapy, a personalized treatment for cancer patients. The process begins at the hospital, where blood is drawn from a cancer patient. The collected blood undergoes a separation process to isolate T cells, a type of white blood cell critical for immune response. The remaining components of the blood are returned to the patient’s body. The isolated T cells are then taken to a laboratory for genetic modification. Here, scientists introduce specific genetic changes to the T cells. These genetic modifications equip the T cells with chimeric antigen receptors (CARs) on their surface. CARs are specialized receptors that enhance the T cells’ ability to recognize and target cancer cells accurately. The genetically modified T cells, now known as CAR T cells, are cultured in the laboratory to increase their number, ensuring there are sufficient cells for effective treatment. Finally, the expanded population of CAR T cells is administered back to the patient through an intravenous injection. These modified cells circulate in the patient’s body, seeking out and destroying cancer cells.

CAR-T therapy is the culmination of extensive research across basic and clinical disciplines. CARs are artificial molecules displayed on cell surfaces, enabling T cells or other effector cells like natural killer (NK) cells to focus their cytotoxic activity on tumor cells expressing the CAR target antigen. These CAR transgenes are introduced into T cells either temporarily or permanently (18, 19). Specifically, using CAR mRNA electroporation leads to temporary CAR expression, whereas employing lentiviral or gammaretroviral gene delivery methods results in the integration of CAR transgenes into T cell genomes, ensuring their stable expression (18, 19). CARs can target either tumor-specific antigens (TSAs) or tumor-associated antigens (TAAs) (20) (**Figure 1**). The capability of CARs to recognize and engage with these target antigens primarily relies on their extracellular domain, comprising a targeting domain and a hinge (or spacer) (21). CARs commonly utilize the scFv from a monoclonal antibody (mAb) as their targeting domain (22). Nevertheless, nanobodies (VHH) and toxins have also been employed for this purpose (23). The hinge serves as the bridge between the extracellular domain and the transmembrane domain of CARs (24). CARs also feature an intracellular domain comprising an activation domain, typically CD3ζ derived from the T-cell receptor (TCR) CD3 complex, and one or two co-stimulatory domains such as CD28, 4-1BB (CD137), ICOS, or OX40 (CD134) (24). The transmembrane domain connects the extracellular and intracellular domains of CARs, serving as an anchor to stabilize CAR molecules within the cell membrane. Upon encountering their target antigen, CAR molecules initiate downstream signaling pathways that activate T cells. This mechanism operates independently of the major histocompatibility complex (MHC) for activation (24). CARs possess the capability to identify target antigens directly, bypassing the need for antigen processing and presentation by MHC molecules on antigen-presenting cells. As a result, any surface antigen primarily expressed on malignant cells, not normal ones, and accessible to targeting with monoclonal antibodies (mAbs), is considered a viable candidate for CAR-T therapy (20, 21).

It hasn’t been long since the first CAR-T product, named tisagenlecleucel, received approval from the US Food and Drug Administration (FDA) in 2017 for clinical use (25, 26). Today, CAR-T therapy stands as an effective treatment option available for patients with certain relapsed or refractory (R/R) hematologic malignancies, including B-cell acute lymphoblastic leukemia (B-ALL), diffuse large B-cell lymphoma (DLBCL), follicular lymphoma (FL), mantle cell lymphoma (MCL), and multiple myeloma (MM) (27).

### 2.2 Success in hematologic malignancies

The development of CAR T-cell therapy for select hematological malignancies represents one of the most remarkable therapeutic advances in the past decade. Currently, CD19-targeted CAR T-cell therapy is approved for relapsed/refractory diffuse large B-cell lymphoma and acute lymphoblastic leukemia (28). However, there is significant interest in the application of CAR T-cell therapy to other hematological malignancies, including multiple myeloma, where the current focus is on the development of B-cell maturation antigen-directed CAR T-cell therapy. Despite the successes achieved to date, there remain significant challenges associated with CAR T-cell therapy and substantial research efforts are underway to develop new targets and approaches.

Presently, CD19 and BCMA represent the predominant targets in CAR-T cell therapy. Despite remarkable success in treating B cell malignancies, relapse following anti-CD19 CAR-T cell therapy and anti-BCMA CAR-T cell therapy is common. Moreover, due to antigenic diversity in acute myeloid leukemia (AML) and the absence of CD19 expression in T cell malignancies, ongoing research is exploring various potential targets. CD19 is a crucial target antigen in B cell malignancies such as B-ALL and NHL. Recent advancements in anti-CD19 CAR-T cell therapy have led to rapid and long-lasting responses in patients with relapsed or refractory (R/R) B-ALL and NHL, fundamentally changing treatment approaches for these conditions. Currently, four anti-CD19 CAR-T cell products have received FDA approval for treating R/R B-ALL and NHL (29). Despite its clinical success, CD19 antigen loss is a common issue (30). To address this, combined therapy using anti-CD19 and anti-CD20 CAR-T cells has been explored for R/R DLBCL, demonstrating safety and feasibility (31). CD22 is prominently expressed on many B cell malignancies, including B-ALL and DLBCL (32, 33). Clinical trials have shown that anti-CD22 CAR-T cell therapy is highly effective in patients with R/R B-ALL and R/R DLBCL who have not responded to previous anti-CD19 CAR-T cell therapy (34–36). Moreover, humanized anti-CD22 CAR-T cells have exhibited potent activity against leukemia cells even with low CD22 expression (37). Emerging strategies in CAR T-cell therapy for B-cell malignancies are focusing on addressing challenges associated with autologous T-cell production, particularly for patients with insufficient healthy T cells. A promising approach involves the use of gene-editing technologies to create universal CAR T cells (UCART19) by modifying donor T cells to introduce a chimeric antigen receptor (CAR) and disrupt T-cell receptor (TCR) and CD52 genes. This process produces “off-the-shelf” CAR T cells capable of evading host immune responses, enabling their use in unmatched recipients. Qasim et al. successfully applied this strategy in two infants with relapsed refractory acute lymphocytic leukemia, achieving molecular remission and bridging them to successful allogeneic stem cell transplantation (38). This groundbreaking application of TALEN-mediated gene editing highlights the potential of universal CAR T cells in treating aggressive B-cell leukemias, offering a scalable and feasible alternative to patient-specific therapies.

Patients diagnosed with relapsed or refractory (R/R) T-cell acute lymphoblastic leukemia (T-ALL) and T-cell lymphomas typically face a grim prognosis. Unlike the notable clinical success of anti-CD19 CAR-T cell therapy in B cell malignancies, the effectiveness and safety of CAR-T cell therapy in T cell malignancies are largely under investigation. A general problem to consider in the production of CAR-T cells is on-target-off-tumor toxicity. This problem is associated with antigens of target expression on normal, non-malignant cells, leading to their destruction by CAR-Ts. Fratricide, i.e. killing of CAR-T cells by each other, is also a problem because the targets of this kind of T cell tumor antigen therapy, antigens expressed on T cells (such as CD5, CD7, etc.), lead to it (39). Other problems are T-cell aplasia and contamination of the CAR T-cell product with tumor cells (39).

CD7 is highly expressed in 95% of T-ALL patients, making it an attractive target for treating T-ALL (40). Two R/R T-ALL patients received allogeneic anti-CD7 CAR-T cell therapy in an open-label, single-arm clinical trial. One patient achieved remission lasting over a year, while the other relapsed 48 days after CAR-T cell infusion (40). Another phase I clinical trial involved 20 R/R T-ALL patients who received donor-derived anti-CD7 CAR-T cell therapy, with 90% achieving complete remission (CR) (41). Additionally, a case study reported the successful treatment of an 11-year-old T-ALL patient, who had not responded to initial treatment, with autologous anti-CD7 CAR-T cell therapy resulting in remission by day 17 and subsequent hematopoietic stem cell transplantation (HSCT) (42). CD5 is expressed in approximately 85% of T-cell malignancies, including T-cell lymphoblastic lymphoma (T-LBL) and peripheral T-cell lymphoma (PTCL). Recent studies have demonstrated the efficacy of anti-CD5 CAR-T cells in eliminating malignant T cells (43). In a phase I clinical trial, a refractory T-LBL patient with central nervous system (CNS) involvement achieved CR within four weeks of receiving anti-CD5 CAR-T cell therapy (44). Moreover, preclinical studies have shown promising activity of anti-CD4 CAR-T cells against T cell malignancies (45). However, targeting CD4, CD5, and CD7 may lead to depletion of normal T cells and fratricide of CAR-T cells, as these antigens are also expressed in normal T cells (46). The chemokine receptor CCR9 is expressed in over 70% of T-ALL patients but in less than 5% of normal T cells. It is associated with multidrug resistance and poor prognosis, making it an ideal target for CCR9-positive T-ALL. Preclinical studies have demonstrated the potent anti-leukemic activity of anti-CCR9 CAR-T cells, which are also resistant to fratricide (47). In addition, several recent clinical trials have shown excellent results for CAR-T cell therapy. In this regard, genome-editing technology can help overcome these problems (39).

AML is the most prevalent acute leukemia in adults. CAR T-cell therapy for AML has been elusive so far, mainly because of the lack of truly AML-specific surface antigens that make targeting AML very challenging. AML cells express various cell surface antigens such as CD123, CD34, and CD33. However, expression of these same antigens is also shared by healthy HSPCs and their myeloid and/or lymphoid progenitors. Besides, production of CAR T cells per se may also present difficulties in patients with active AML, possibly because of the inhibition of T-cell expansion by AML blasts, or previous exposure to chemotherapy damaging T cells (48). However, progress has been made toward the use of CAR T-cell therapy in this disease.

Recently, CLL-1, LILRB4, and Siglec-6 have emerged as potential targets. In preclinical studies, CLL-1, a myeloid cell surface marker overexpressed on leukemic stem cells, has shown specificity in eliminating CLL-1-positive leukemia (49–51). Notably, CLL-1 is absent in hematopoietic stem cells, enhancing its therapeutic potential. NPM1 mutations are present in 30%-35% of AML cases and are considered pivotal in leukemic cell initiation. CAR-T cells targeting a nucleophosmin neoepitope, presented by HLA-A2, demonstrated potent anti-leukemia effects in preclinical models (52). CD70, expressed on AML blasts but not normal myeloid cells, is also being investigated as a promising CAR-T cell therapy target (53, 54). LILRB4, highly expressed in monocytic AML cells, presents another attractive target for monocytic AML (55). Siglec-6, found in approximately 60% of AML patients and absent on normal hematopoietic stem and progenitor cells, has effectively eliminated AML blasts in preclinical xenotransplantation models. These findings support Siglec-6 as a validated target for CAR-T cell therapy in AML (56). A novel approach to CAR T-cell therapy for AML aims to overcome the challenge of prolonged myeloablation while ensuring long-term persistence of therapeutic cells. This strategy involves gene editing to remove the CAR target antigen, such as CD33, from donor hematopoietic stem and progenitor cells (HSPCs). After these CD33−/− HSPCs are transplanted into the patient and engrafted, CD33-specific CAR T cells from the same donor can be administered, allowing normal hematopoiesis to continue without being targeted by the CAR T cells (48). Early studies using CRISPR/Cas9 technology have shown that CD33−/− HSPCs can resist CD33-directed CAR T cells while maintaining normal hematopoietic and immune functions (57). A clinical trial at the University of Pennsylvania is being developed to test this approach in patients with relapsed/refractory AML. This strategy not only highlights the potential of gene editing in improving CAR T-cell therapies but also opens new avenues for targeting other antigens, such as CD123, with careful consideration of their biological roles and potential impact on healthy tissues.

### 2.3 Challenges in treating solid tumors

CAR-T cell therapy, while highly effective in treating certain cancers, is associated with a range of adverse effects that can significantly impact patient outcomes. These include immune-related toxicities such as Cytokine Release Syndrome (CRS) and Immune-Effector-Cell-Associated Neurotoxicity Syndrome (ICANS), both of which can cause severe inflammatory and neurological complications. Metabolic toxicities like Tumor Lysis Syndrome (TLS) are also common, arising from the rapid destruction of cancer cells, which can lead to life-threatening metabolic imbalances. Additionally, on-target/off-tumor toxicity occurs when CAR-T cells attack healthy tissues that express the same antigen as the cancer cells, leading to potential organ damage. These toxicities are influenced by various factors, including the patient’s health status, tumor burden, the dose of CAR-T cells, and the rate of infusion, necessitating careful monitoring and tailored management strategies to mitigate risks and enhance the safety and efficacy of CAR-T cell therapy.

#### 2.3.1 Immune-related toxicities

The most serious and prevalent toxicity associated with CAR-T cell therapy is a systemic inflammatory reaction known as cytokine release syndrome (CRS) (58). Activation of CAR-T cells upon recognition of tumor antigens triggers CRS, characterized by severe systemic inflammation. Following CAR-T cell infusion, there is a significant increase in serum levels of cytokines such as interferon-gamma (IFN-γ), interleukin-6 (IL-6), tumor necrosis factor-alpha (TNF-α), granulocyte-macrophage colony-stimulating factor (GM-CSF), IL-2, IL-8, and IL-10, leading to a cytokine storm. This initial response is followed by a secondary inflammatory phase involving antigen-presenting cells (APCs) like dendritic cells (DCs), B cells, macrophages, and monocytes, which express the cell surface protein CD40. Activated CAR-T cells also express high levels of the CD40 ligand (CD40L) (59).

Patients should be closely monitored from the emergence of early symptoms of CRS and treated symptomatically with antipyretics and analgesics, although it is contraindicated for NSAIDs because they can alter kidney function. If a diagnosis of an infection is made, particularly in patients who are febrile and neutropenic, they should be ruled out and started on empiric antibiotics, taking into consideration the raised incidence of infection post a regimen that deploys lymphocytes to such a great extent. Studies show that almost one-quarter of patients under CD19-targeted CAR T-cell therapy experience infections, mostly bacteremias and respiratory viral infections, in the first four weeks post-infusion. The use of prophylactic antibiotics is not universally established but is done in some centers (60). Other supportive measures for CRS include antiemetics, oxygen, intravenous fluids, and low-dose vasopressors if needed; it generally avoids corticosteroids (60). Prompt recognition and treatment of severe CRS (sCRS) is important as it may lead to multiorgan failure akin to septic shock. First-line therapy of sCRS is tocilizumab, an IL-6 receptor antagonist, with a high response rate. Next steps in failure cases include the use of corticosteroids, whereas other inflammatory cytokine-targeted therapies, such as anti-TNFα or IL-1R inhibitors, should be offered in rare, resistant cases (60).

Immune-effector-cell-associated neurotoxicity Syndrome (ICANS), formerly known as CAR-T-cell-related encephalopathy syndrome, represents the second most common adverse effect of CAR-T cell therapy. ICANS is characterized by cytokine-mediated neurotoxicity rather than direct cytotoxic effects, although its exact pathophysiology remains unclear. Research suggests that endothelial activation plays a role in the development of ICANS (61). Endothelial activation associated with CRS can disrupt the blood-brain barrier (BBB), allowing inflammatory cytokines and immune cells to infiltrate the cerebrospinal fluid and reach the central nervous system (CNS). Once in the brain parenchyma, these cytokines and immune cells can cause inflammation, leading to neuronal impairment or damage. CAR-T cells, along with monocytes and macrophages, are attracted to the CNS and contribute to the release of cytokines, which are central to the development of ICANS (59). Pericytes, a type of mural cell surrounding capillary endothelium and expressing CD19, are crucial for maintaining the integrity of the BBB. In treatments targeting CD19, increased BBB permeability due to pericyte activation has been implicated in ICANS development (62). ICANS manifests with various neurological symptoms that often progress in a characteristic pattern. Symptoms can appear as early as the fourth or fifth day after CAR-T cell infusion and as late as the third or fourth week (63, 64). ICANS rarely occurs without preceding CRS, and when it does, it tends to be mild (65). Common symptoms include headache, tremors, speech difficulties, confusion, delirium, impaired consciousness (such as obtundation, lethargy, and stupor), and occasionally, focal neurological deficits (66).

Prompt recognition and management of CNS toxicity in CAR T-cell therapy are crucial, similar to the approach for CRS. Patients experiencing neurotoxicity require close monitoring, with ICU transfer recommended for those with grade ≥3 toxicity and considered for those with grade 2 toxicity, depending on the center’s policy. In severe cases, neurologic toxicity may necessitate intubation and mechanical ventilation for airway protection, even without respiratory failure. Notably, fever within the first 36 hours post-infusion has been associated with a high sensitivity for subsequent severe neurotoxicity. Management typically involves corticosteroids, tailored to the severity and specific CAR T-cell product used. Importantly, unlike CRS, neurotoxicity generally does not respond to tocilizumab, and in some cases, the drug may even exacerbate the condition. Additionally, neurotoxicity often resolves more slowly than CRS (60).

#### 2.3.2 Metabolic toxicities

When cancer treatment effectively kills cells, it can release significant amounts of phosphate, potassium, and nucleic acids into the bloodstream, potentially leading to tumor lysis syndrome (TLS) (67). While TLS has traditionally been associated with chemotherapy, CAR-T cell therapy has also been linked to acute anaphylaxis and TLS, sometimes occurring even without prior conditioning chemotherapy (68, 69). The rapid death of lymphoma cells following CAR-T cell treatment can pose challenges if the kidneys are unable to process the byproducts of cell lysis quickly enough, resulting in conditions such as hyperuricemia, hyperkalemia, hyperphosphatemia, and hypocalcemia. Accumulation of uric acid, calcium phosphates, and ferritin can further exacerbate acute kidney injury, leading to systemic inflammation and iron overload.

Unlike other novel therapies for hematologic malignancies that have heightened the risk of TLS, TLS is relatively uncommon following CAR T-cell therapy, even in high-risk scenarios. Nevertheless, precautionary measures, such as intravenous hydration and prophylactic administration of allopurinol or febuxostat, should be implemented prior to starting conditioning lymphodepleting chemotherapy, particularly in patients with elevated uric acid levels or high tumor burden. It is essential to closely monitor for signs and symptoms of TLS and manage them according to established guidelines to prevent complications (60).

#### 2.3.3 On-target/off-tumor toxicity

**Figure 2:**
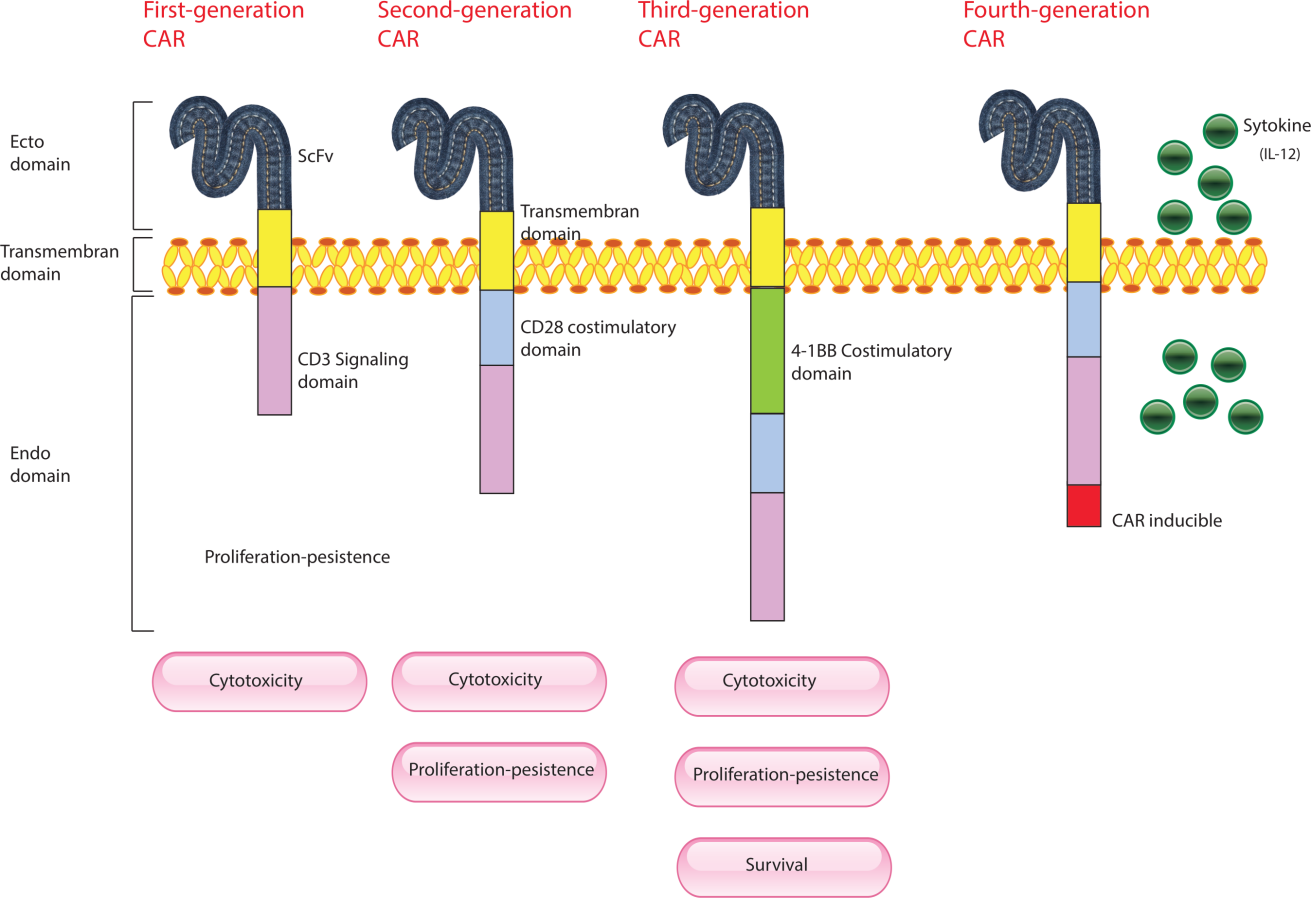
Evolution of CAR-T Cells. This figure illustrates the development of CAR-T cells through three generations, each incorporating advancements to improve their effectiveness against cancer. First Generation CAR-T Cells: These are composed of a single-chain variable fragment (scFv) derived from an antibody, which is responsible for targeting cancer cells, and the CD3 immunoglobulin, which is part of the T-cell receptor complex essential for initiating T-cell activation. Second Generation CAR-T Cells: Building on the first generation, these cells include additional co-stimulatory molecules, such as CD28. This enhancement provides stronger and more sustained activation signals to the CAR-T cells, improving their persistence and effectiveness in targeting and destroying cancer cells. Third Generation CAR-T Cells: These cells incorporate multiple co-stimulatory molecules, such as CD28, CD134 (OX40), and CD137 (4-1BB). The inclusion of multiple stimulatory signals further enhances the CAR-T cells’ ability to proliferate, survive, and eliminate cancer cells, offering an even more robust therapeutic effect.

Ideally, CAR-T cell therapy targets antigens expressed exclusively on malignant cells, sparing healthy tissues. However, solid tumors have seen limited success with this approach. Many tumor antigens are tumor-associated antigens (TAAs), present on both healthy and tumor cells. Consequently, CAR-T cells often struggle to differentiate normal cells from cancerous ones, resulting in “on-target, off-tumor” toxicity (59). This phenomenon is more common in solid tumors, underscoring the need for extensive research into identifying tumor-specific antigens (TSAs). Several cases illustrate the challenges posed by TAAs expressed on normal tissues. For instance, the first patient treated with CAR-T cells targeting HER2 experienced respiratory distress and significant lung infiltrates within 15 minutes of infusion, leading to lung damage and death (70). Similar lung toxicity was observed in clinical trials testing CAR-T cells targeting CEA (71). It’s important to recognize that the occurrence of adverse effects in CAR-T cell therapy can vary significantly depending on the specific CAR-T product (**Figure 2**), cancer type, and individual patient factors.

Thus, it is important to prevent “on-target/off-tumor” effects, which would result in collateral damage to normal tissues with CAR T-cell therapy. This can be achieved by targeting antigens that are more selective, such as κ and λ light chains of immunoglobulins to preserve humoral immunity, potentially maintaining antitumor activity in some B-cell malignancies (72, 73). Another approach is the extinction of the target antigen on the normal population of hematopoietic stem cells. This has been demonstrated in a study where CD33-deficient stem cells were genetically modified and co-infused with CD33+ CAR T cells. The infused T cells engrafted, allowing for normal myeloid function with no evidence of off-target effects. Furthermore, optimization of the CAR design, ranging from engineering dual CARs for multi-antigen recognition of tumor-specific antigens to the use of affinity-tuned CARs, may increase the precision of target recognition and minimize the risk of relapse from antigen deletion (74, 75). Lastly, the capacity for a targeted reduction of CAR activity in the event of severe toxicity is an active research area, which may be achieved using techniques such as inducible suicide genes, monoclonal antibodies, small molecule modulators, or CRISPR/Cas9 technology to remotely control or transiently turn off CAR T cells (61).

#### 2.3.4 Other factors influencing toxicity

*In vivo*, the expansion of CAR-T cells and associated toxicity may be exacerbated by factors such as tumor burden, intensity of conditioning therapy, higher infusion doses, and CAR design. For example, pediatric patients with high baseline tumor burdens in B-ALL tend to experience more pronounced CAR-T cell proliferation and more severe cytokine release syndrome (CRS) (59). Clinical studies have consistently shown that patients with larger tumor burdens experience more severe and frequent CRS, likely due to heightened T cell activation levels (76, 77). Moreover, patients with higher initial burdens of ALL and those receiving higher doses of CD19 CAR-T cells have been found to have increased incidence rates of CRS (78, 79). Improving CAR-T cell efficacy and durability of response is the central goal of conditioning therapy aimed at enhancing clinical outcomes in cancer patients. Even without chemotherapy conditioning, adverse effects such as thrombocytopenia, anemia, and neutropenia have been documented (59). Effective strategies for prevention and mitigation of these toxicities include preemptive treatments, careful patient monitoring, and adjustments in CAR-T cell administration protocols. Understanding and managing these adverse effects is crucial for maximizing the therapeutic benefits of CAR-T cell therapy while minimizing its risks.

Recently, CAR-Natural Killer (CAR-NK) and CAR-macrophages (CAR-M) were introduced as a complement/alternative to CAR-T cell therapy for solid tumors. CAR Natural Killer (NK) cells have several advantages over CAR T cells as the NK cells can be manufactured from pre-existing cell lines or allogeneic NK cells with unmatched major histocompatibility complex (MHC); can kill cancer cells through both CAR-dependent and CAR-independent pathways; and have less toxicity, especially cytokine-release syndrome and neurotoxicity. At least one clinical trial showed the efficacy and tolerability of CAR NK cell therapy. Additionally, CAR-NK cells might be generated in large scale from several sources which would suggest them as promising off-the-shelf product. CAR-M immunotherapy with its capabilities of phagocytosis, tumor-antigen presentation, and broad tumor infiltration, is currently being investigated (80, 81).

In contrast, CAR-Natural Killer (CAR-NK) and CAR-Macrophages (CAR-M) have recently been introduced as alternatives or complements to CAR-T cell therapy for solid tumors. There are several advantages of CAR NK cells over CAR T cells: NK cells can be manufactured from pre-existing cell lines or allogeneic NK cells with unmatched major histocompatibility complex. They can kill cancer cells through both CAR-dependent and CAR-independent pathways, and have less toxicity, particularly when it comes to cytokine-release syndrome and neurotoxicity. At least one clinical trial has shown the effectiveness and safety of CAR NK-cell therapy. Furthermore, CAR-NK cells can be derived on a large scale from various sources, making them a potential off-the-shelf product. Currently, CAR-M immunotherapy, which involves phagocytosis, tumor antigen presentation, and extensive tumor infiltration, is under investigation. In this study, we designed an adenovirus-induced CAR-M using an anti-HER2 CAR and the CD3ζ intracellular domain. This CAR-M demonstrated *in vitro* specificity in terms of antigen-specific phagocytosis against HER2-positive tumor cells. A single injection of anti-HER2 CAR-M reduced tumor load and prolonged survival in mice. It also shifted M2 macrophages into M1 macrophages, stimulated an inflammatory tumor microenvironment (TME), and exhibited anti-tumor cytotoxicity. Importantly, HER2 CAR-M had the capability of inducing epitope spread, which could be an additional approach to prevent tumor immune escape. Another study combined anti-HER2 CAR with transduced primary human CD14+ peripheral blood monocyte-derived macrophages. These CAR-Ms stimulated phagocytosis of the HER2+ ovarian cancer cell line SKOV3 in a dose-dependent manner. The authors also demonstrated that the transduction of macrophages was not impaired by the antitumor activity, as their transduction with a control CAR did not exhibit any antitumor activity. *In vivo*, the SKOV3 tumor burden in NOD-SCID mice was significantly reduced in those cohorts that had been treated with primary human anti-HER2 CAR-Ms. The authors further showed that the CAR-Ms persisted and remained resistant to the immunosuppressive cytokines secreted by the TME. In contrast, the CAR-Ms secreted pro-inflammatory cytokines, leading to the conversion of macrophages from the M2 to the M1 phenotype and subsequently transforming the TME into a proinflammatory environment. Additionally, the combination of donor-derived T cells with CAR-Ms enhanced the antitumor response *in vivo*. When murine-derived anti-HER2 CAR-Ms were infused, Pierini et al. reported inhibition of tumor growth, extended overall survival, increased levels of CD4+ and CD8+ T cells, NK cells, and dendritic cells in the TME. These researchers also found that CAR-Ms play a crucial role in regulating the TME through the upregulation of MHC I/II expression on cancer cells.

## 3 CRISPR/Cas9 technology

### 3.1 CRISPR/Cas9: advantages of CRISPR/Cas9 over traditional methods

Genome editing involves modifying genomic DNA to artificially alter genetic information, resulting in permanent changes to the function of the targeted gene (82). Several tools have been developed for precisely modifying specific regions of the genome. Genome editing nucleases, such as zinc finger nucleases (ZFNs), meganucleases, and transcription activator-like effector nucleases (TALENs), induce double-strand breaks at specific genomic sites (83). These nucleases facilitate targeted modifications by initiating endogenous DNA repair mechanisms, primarily non-homologous end joining (NHEJ), which repairs double-strand breaks without requiring a template (84). This approach can effectively replace or delete target genes, although designing and engineering these nucleases to target new sequences remains a significant challenge (83, 85).

**Figure 3:**
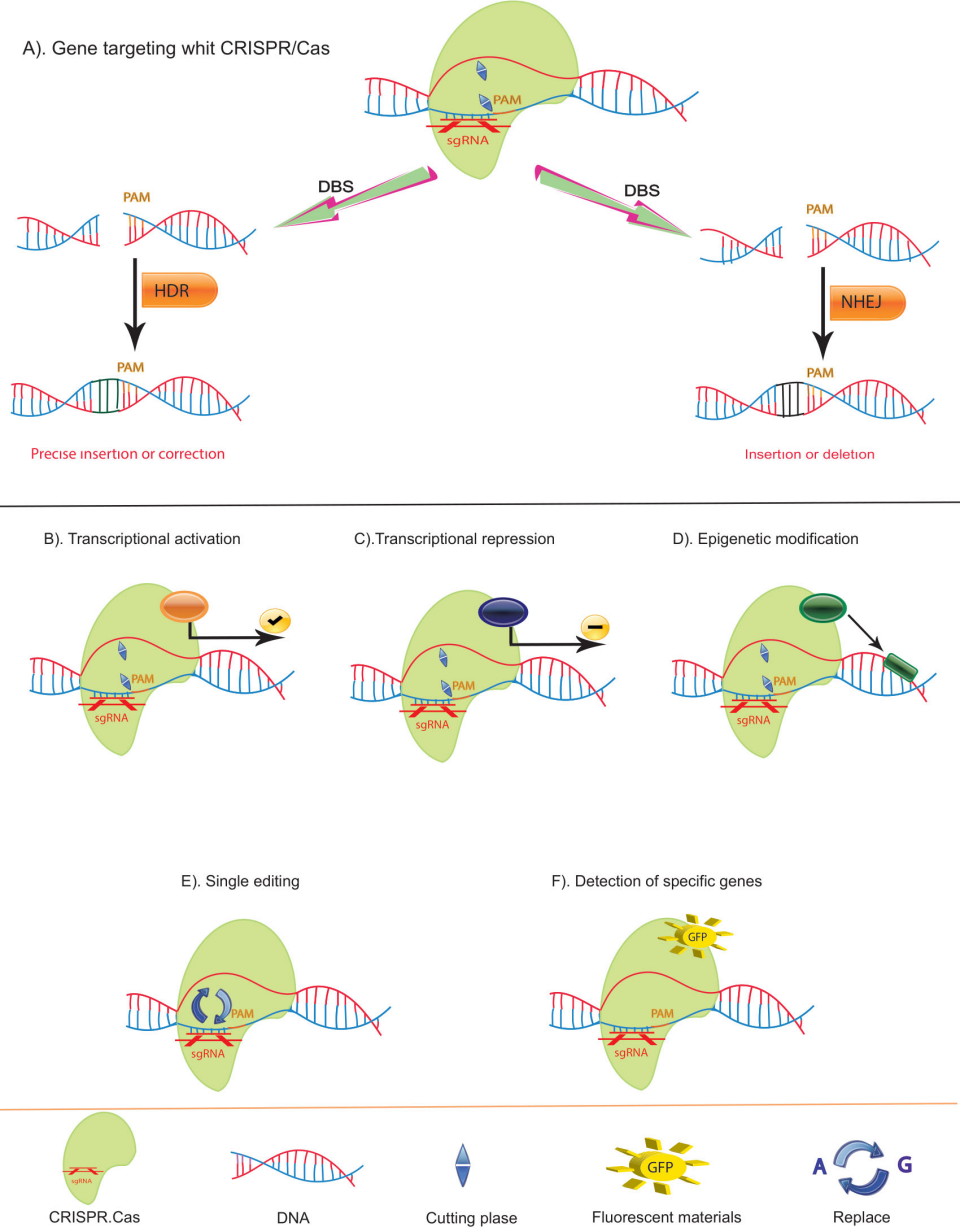
Function of CRISPR/Cas and Variants. **(A)** Double-Strand Break (DSB) Repair Mechanisms Using CRISPR/Cas: The CRISPR/Cas system can induce DSBs, which are repaired by two primary pathways: Non-Homologous End Joining (NHEJ) and Homology-Directed Repair (HDR). NHEJ often results in small insertions or deletions, leading to disruptive frameshift mutations and premature stop codons, making it ideal for gene knockouts or generating point mutants. In contrast, HDR enables precise mutations. **(B)** Cas9-VP64 Fusion: Cas9 can be fused with the VP64 transcriptional activator (yellow) to activate gene transcription by binding upstream of the transcription start site. **(C)** Cas9-KRAB Fusion: Cas9 can also be fused with the KRAB repressor (red) to downregulate gene transcription by binding to the transcription start site. **(D)** Cas9-Epigenetic Modifier Fusion: Cas9 can be linked with epigenetic modifiers (black) to alter local methylation patterns, thereby modifying gene expression epigenetically. **(E)** Cas9-Base Editors: By fusing Cas9 with base editors (purple), precise single nucleotide exchanges can be achieved without causing DSBs. **(F)** Fluorescent Cas9: If the cutting function of Cas9 is deactivated and it is equipped with a fluorescent marker, it can bind to specific DNA sequences, creating a green fluorescent signal. This enables the identification and visualization of specific sequences.

The CRISPR/Cas9 system has emerged as a leading gene editing tool in recent years (**Figure 3**). Initially discovered in bacteria as a defense mechanism against viruses, this system consists of an endonuclease (Cas9) and a single-guide RNA (sgRNA) that directs Cas9 to specific locations in the genome through base pairing. Cas9 then cleaves the target DNA, prompting the host cell to repair the break. If a donor template with homologous arms is present, homology-directed repair (HDR) can occur, resulting in precise editing of the genome. Alternatively, non-homologous end joining (NHEJ) can resolve the break by inserting or deleting nucleotides (indels), often disrupting the gene’s reading frame. This system is highly effective, simple to use, and widely applicable, making it a powerful tool for genome editing (86).

Compared to previous genome editing tools like TALEN and ZFN, CRISPR/Cas9 offers advantages such as rapid, cost-effective sgRNA production, in contrast to the synthesis of custom guide proteins for TALEN or ZFN. Additionally, CRISPR/Cas9 can simultaneously modify multiple genes by utilizing multiple sgRNAs targeting different genomic sites, surpassing the capabilities of ZFN and TALEN (87, 88). This system also excels in its ability to alter the epigenome, transcriptome, and genome of immune-related cells and cancer cells (89). The application of CRISPR/Cas in cancer treatment hinges on the careful selection of target genes (90), offering innovative solutions for clinical applications in cancer immunotherapy and gene therapy. Nonetheless, ongoing research is essential to further refine and optimize the CRISPR/Cas method for genome editing.

### 3.2 Applications in cancer research

In cancer research, the applications of CRISPR-Cas9 mainly involve the screening of oncogenic mutations and tumor suppressors, the construction of *in vivo* and *in vitro* cancer models, and cancer gene therapy (91). With a relatively high editing efficiency and few off-target effects, the CRISPR-Cas gene-editing system is able to change the biological behavior of tumor cells from the level of the genome, reduce the destruction of normal human tissue cells, and increase the survival time of patients.

Genome-wide CRISPR-Cas9 screening has produced numerous high-quality data (92, 93). Sidi Chen et al. obtained specific functionally defective mutations essential for tumor growth and metastasis using genome-wide CRISPR screening, such as Cdkn2a, Fga, and Cryba4 (94). Similarly, Ryan d. Chow et al. identified several functional suppressors and the cooccurrence and correlation of specific mutations in glioblastoma through *in vivo* CRISPR screening. They identified cooccurring driver combinations including B2m-Nf1, Mll3-Nf1 and Zc3h13-Rb1 by commutation analysis (95).

It is essential to develop models that accurately reflect the disease to better study cancer evolution and pathogenesis. For this reason, CRISPR-Cas9, with its precise gene-editing technology, is considered one of the game-changers employed to develop relevant models of cancer that best imitate human tumors. These models can be broadly divided into two categories: *in vitro* and *in vivo*. *In vitro* methods include organoid technology, which recapitulates tumor behavior via the introduction of loss-of-function mutations by knocking out or knocking down selected genes. Additionally, *in vivo* models would likely be established by introducing CRISPR-Cas9 specifically to edit oncogenic mutations or chromosomal rearrangements in tissues to further explain cancer biology thoroughly (96).

To effectively deliver CRISPR components into target cells, various systems have been developed, primarily categorized into viral-based and nonviral methods. In cancer research, viral-based delivery systems such as adeno-associated virus (AAV), lentivirus, and adenovirus are commonly used for the plasmid-based CRISPR-Cas9 system. AAV, in particular, stands out due to its small, non-enveloped single-stranded DNA structure derived from the non-pathogenic parvovirus family. It has gained attention for its minimal immunogenicity and ability to maintain gene expression in non-dividing cells, making it a promising tool for gene delivery. Additionally, AAV can serve as a donor template in homologous recombination, facilitating DNA strand exchange between similar sequences (97, 98). Although AAV vectors are not yet in clinical trials, they hold significant potential for future therapeutic applications. On the other hand, nonviral delivery systems, including hydrodynamic injection, electroporation, nanoparticles, and transposon carriers, offer greater safety despite being generally less efficient than viral vectors (96). A major challenge with nonviral methods is their lack of tissue specificity, an issue that may be mitigated by modifying the Cas9 protein accordingly.

CRISPR-based genetic and epigenetic manipulation of immune responses has emerged as a promising strategy in immunotherapy for combating cancer initiation and progression. This approach involves enhancing host immunity at specific genetic loci, boosting tumor immunogenicity, and overcoming tumor immune evasion mechanisms. Thus far, modifying immune cells ex vivo to suppress immune checkpoint expression or to introduce synthetic immune receptors, such as chimeric antigen receptors (CARs), has demonstrated efficacy in treating certain cancers like melanoma, lymphoma, liver, and lung cancer (99).Besides the success of the CRISPR-Cas9 system, the development of a nuclease-deactivated Cas9 (dCas9) variant has expanded CRISPR technologies to include epigenome engineering. By introducing two mutations—D10A and H840A—into Cas9, the wild-type system is converted into an inactivated cleavage capacity but retains RNA-guided DNA-binding specificity (100). As was first shown with the engineered zinc finger proteins, dCas9 can be fused to a variety of effector domains that enable highly targeted and tunable transcriptional activation or repression, editing of epigenetic marks, or fluorescent tagging of endogenous genes without direct genomic modification (99).

In the area of oncology, these dCas-based tools have shown strong activation of tumor suppressor genes, such as PTEN, in breast cancer and melanoma; MASPIN, in breast and lung cancers; REPRIMO, in breast and gastric cancers; SARI, in colon cancer; and DKK3, in prostate cancer. Successful suppression of oncogenes was also attained using dCas9 in colon cancer, targeting BRAF, HER2, and MYC; pancreatic cancer, targeting KRAS; and liver cancer, targeting GRN (99). Also, several works describe that epigenome editing can be very efficient, as it has attained almost complete gene repression or robust (several-fold) gene activation with low off-target effects, which mostly depend on the effector domains’ nature used (100, 101). Finally, in contrast to genome engineering by Cas9, which unavoidably leads to permanent changes, epigenetic approaches result in reversibility and thereby bypass the risk of inducing sequence changes in the target DNA—a most crucial factor when it comes to targeting tumors with high degrees of genetic instability (99). In addition, the durability of such epigenetic and transcriptional changes that are induced by dCas9 editing might depend on the specific combination of effectors or on targeted loci. Therefore, current research in epigenome engineering will have to focus on further fine-tuning the technology for the manipulation of different loci within diverse cell types with differing chromatin microenvironments.

## 4 Synergistic potential of CAR-T and CRISPR/Cas9

CRISPR/Cas9 technology has been extensively tested across various cell types and organisms. It has played a pivotal role in advancing CAR-T cell development and enhancing other genome editing tools (102). Notably, CRISPR/Cas9 modifications are currently under investigation in clinical trials aimed at improving CAR-T cell therapy (**Table 1**). Before the advent of CRISPR/Cas9, other genome editing methods like zinc finger nucleases (ZFNs) or TALENs were employed. However, CRISPR/Cas9 has surpassed these methods in terms of cost-effectiveness and practicality (103, 104). Moreover, it enables multiplex gene editing, a significant capability that facilitates the generation of universal CAR-T cells by long-term silencing of endogenous TCR, HLA class I molecules, and inhibitory checkpoints such as CTLA4 and PD1 (102–104). Research has proved that employing CRISPR/Cas9 to delete PD-1 resulted in enhanced long-term persistence and activity of CAR-T cells (preclinical study) (105), and similarly, the deletion of CTLA-4 using CRISPR/Cas9 improved the proliferation and activity of CAR-T cells (preclinical study) (106). Additionally, CRISPR/Cas9 provides a robust alternative to conventional lentiviral insertion of CARs by avoiding random genome integration and uncontrolled construct expression (107).

**Table 1:** Clinical trials of engineering CAR-T cells based on CRISPR/Cas9 technology.

NCT ID	Cancer	Target antigen	CART Cell	Patients (n)	Phase	Advantages
**NCT04637763**	Non-Hodgkin Lymphoma	CD19	CD19-CAR-T	72	1	Broaden applicability
**NCT04502446**	T cell malignancy, Diffuse Large B-Cell Lymphoma	TCR and MHC I KO via CRISPR/Cas9	CD70-CAR-T	26	1	–
**NCT04035434**	B-cell Lymphoma, B-cell ALL	CD19	CD19-CAR-T	227	1/2	Cost reduction
**NCT04244656**	Multiple Myeloma	–	BCMA-CAR-T	26	1	Enhance effector function
**NCT03166878**	Leukemia, Lymphoma	TCR and B2M (knock out)	CD19-CAR-T	80	1/2	Broaden applicability
**NCT03398967**	Leukemia, Lymphoma	TCR and B2M (knock out)	CD19/CD20/CD22-CAR-T	80	1/2	Cost reduction Broaden applicability
**NCT04037566**	ALL, Lymphoma	HPK1 (knock out)	CD19-CAR-T	40	1	Cost reduction Enhance effector function
**NCT04438083**	Renal Cell Carcinoma	–	CTX130	107	1	–
**NCT03747965**	Multiple Solid Tumors	PD-1 (knock out)	Mesothelin CAR-T	10	1	Enhance effector function
**NCT05795595**	Multiple Solid Tumors	–	CD70-CAR-T	250	1/2	–
**NCT03545815**	Solid Tumors	PD-1 and TCR (knock out)	Mesothelin CAR-T	10	1/2	Cost reduction Broaden applicability Enhance effector function
**NCT05812326**	Breast Cancer	PD-1 (knock out)	MUC1-CAR-T	15	1/2	

Data extracted from https://clinicaltrials.gov/. B2M, β-beta 2-microglobulin; CD, cluster of differentiation; HPK1, hematopoietic progenitor kinase 1; PD-1, programmed cell death protein 1; TCR, T- cell receptor.

Some researchers have proven that, in terms of effectiveness, persistence, and reducing side effects, autologous CAR-T cells perform better than allogeneic CAR-T cells. This has prompted researchers to further optimize allogeneic CAR-T cells. Li et al. used CRISPR/Cas9 technology to knock out T-cell receptor (TCR) and HLA-I/II genes in CAR-T cells, introducing exogenous HLA-E expression to improve CAR-T cell persistence and prevent rejection in preclinical studies. A more promising approach being pursued is the development of a stable supply of universal allogeneic CAR-T cells for cancer therapy by generating iPSCs, which have virtually unlimited replicative potential and broad differentiation abilities. Wang et al. demonstrated that using CRISPR/Cas9 to integrate the CAR gene into the endogenous TCRα constant (TRAC) locus in iPSCs results in CAR-T cells with lower immunogenicity, enhanced tumor cytotoxicity, improved survival, and reduced allogeneic response risk. Furthermore, Ueda et al. enhanced these iPSC-derived CAR-T cells by editing genes with CRISPR/Cas9 to delete the diacylglycerol kinase gene and introduce IL-15 and its receptor subunit, leading to improved proliferation and increased longevity in preclinical studies.

Despite its advantages, the CRISPR/Cas9 system is not without limitations. Off-target effects can occur, potentially affecting cell fitness. However, several strategies, such as precise sgRNA design, truncated sgRNAs, chimeric DNA-RNA-based sgRNAs, and the use of different Cas9 variants, have been developed to mitigate these effects (108–111). Another limitation is the low frequency of homology-directed repair (HDR), which limits the efficiency of gene correction and addition. Enhancers of HDR and inhibitors of non-homologous end joining (NHEJ) can be employed to promote these processes (112–114). Despite these challenges, overcoming these limitations is crucial for generating effective CAR-T cells (**Table 2**).

**Table 2:** Enhanced techniques and strategies for effective gene editing in CAR-T cells.

CAR-T cell	Target gene (s)	Cas9 delivery system	Gene-editing efficiency	Multiplex Efficiency	Antitumor Activity	Disadvantages	Ref
CD19-CAR-T cells PSCA-CAR-T cells	B2M PD1 TRAC TRBC	mRNA	50% NHEJ (multiplex) 70–90% NHEJ (single gene disruption)	Enhanced *in vivo* antitumor activity	Yes, *in vitro* and animal models	Multiplex gene editing led to reduced overall editing efficiencies.	(115)
CD19-CAR-T cells	LAG3	RNP	70% NHEJ (single gene Disruption)	Not applicable	Robust antigen-specific antitumor activity in cell culture and murine model	The efficiency of gene editing has room for further optimization.	(116)
GPC3-CAR-T cells	PD1	RNP	85% NHEJ (single gene disruption)	Not applicable	Enhanced *in vivo* antitumor activity, improved persistence and infiltration	Not applicable	(117)
139 CAR-T cells	DGKα DGKζ	RNP	60–70% NHEJ (single gene disruption)	Not applicable	Significant regression of tumors in a xenograft mouse model	The efficiency of gene editing has room for further optimization.	(118)
CD7-CAR-T cells CD19-CAR-T cells	CD7 TRAC	DNA plasmid	70 NHEJ (multiplex) 90% NHEJ (single gene disruption)	Not applicable	Efficacy *in vitro* and *in vivo* without induction of xenogeneic GvHD	Not applicable	(119)
CD19-CAR-T cells PSCA-CAR-T cells	TCR, HLA class I, Fas, PD1, CTLA4	lentivirus -based one-shot system/mRNA/RNP/mRNA	50% NHEJ (multiplex) and 90% NHEJ (single gene disruption) 82% NHEJ (single gene disruption) 76% NHEJ (single gene disruption)	Simultaneous gene editing of four loci attempted	Enhanced antitumor activity against multiple inhibitory pathways	Multiplex gene editing led to reduced overall editing efficiencies. The limited packaging capacity of lentiviral vectors led to reduced gene-editing efficiency.	(120)

B2M, β-2-microglobulin; CTLA4, cytotoxic T-lymphocyte-associated protein 4; DGKα/ζ, diacylglycerol kinase α/ζ subunit; GPC3, glypican 3; LAG3, lymphocyte activation gene 3; NHEJ, non-homologous end joining; PD1, programmed cell death protein 1; PSCA, prostate stem cell antigen; RNP, ribonucleoprotein; TRAC/TRBC, T-cell receptor α/β constant subunit.

To address issues such as targeting healthy cells and committing fratricide—an on-target off-tumor effect—rational selection of chimeric antigen receptors (CARs) is essential (121). In T-cell-derived malignancies, identifying suitable tumor-associated antigens (TAA) is particularly challenging, as many TAAs are shared between malignant T cells and CAR-T cells (122). To prevent self-destruction, researchers have disrupted the expression of widely expressed T-cell antigens in CAR-T cells. Specifically, clusters of differentiation 3, 5, and 7 (CD3, CD5, and CD7) have been successfully targeted using gene-editing technologies such as transcription activator-like effector nucleases (TALENs) and CRISPR/Cas9. The resulting CAR-T cells demonstrated significant antitumor efficacy while reducing the risk of fratricide, offering a promising approach to improving CAR-T cell therapies in T-cell malignancies (123–125). Pinz et al. (45). developed CD4-CAR-T cells to treat peripheral T-cell lymphomas but encountered significant fratricide, resulting in the enrichment of CD4+ CD8+ CD4-CAR-T cells. To maintain a stable CD4/CD8 CAR-T cell ratio, investigating the impact of a CD4 knockout could be beneficial. Similarly, to address the potential fratricide of anti-CD319 CAR-T cells in multiple myeloma treatment, Galetto et al. utilized TALENs technology to inactivate CD319, which is broadly expressed in activated T cells. This approach successfully prevented the loss of CD319-positive CAR-T cells during T-cell expansion, highlighting a promising strategy to mitigate fratricide in CAR-T cell therapies (126).

CRISPR/Cas9 delivery into T cells can be achieved through various methods, including plasmid DNA, messenger RNA (mRNA), or delivery as ribonucleoproteins (RNPs). The efficiency of gene editing varies significantly depending on the cell type and donor, but both mRNA and RNP delivery methods have shown promise in achieving high rates of insertion or deletion mutations (indels) (127). This variability in efficiency underscores the importance of choosing the appropriate delivery method to ensure accurate editing of CAR-T cells.

Traditional CAR-T cell production relies on viral transduction of CARs, but concerns over potential side effects from integrative viruses have prompted the exploration of alternative methods. One preferred approach by several research groups involves targeted transgene integration into the TRAC gene using the CRISPR/Cas9 system. This method achieves high integration rates by delivering the repair template through AAV6 transduction or long single-stranded DNA (ssDNA) electroporation (128, 129). Another delivery method utilizes integrase-defective lentiviruses (IDLVs), known for their large genome capacity, low pathogenicity, and ability to transduce both dividing and non-dividing cells (130, 131). Despite these advantages, no preclinical trials utilizing IDLVs have been conducted thus far.

## 5 Integration of CAR-T and CRISPR/Cas9 in clinical settings: analysis of outcomes

CAR-T cell therapy stands out as a highly promising treatment option for refractory hematologic malignancies. Over the years, significant advancements have transformed the design of CARs (132). These include incorporating features such as coexpression of costimulatory molecules, cytokines, and suicide genes to enhance both efficacy and safety (133). Furthermore, the repertoire of tumor targets for CAR-T cells has expanded beyond CD19 to encompass a wide array of new targets. These include CD20, CD22, CD30, CD33, CD138, CD171, CEA, epidermal growth factor receptor (EGFR), EGFRvIII, ErbB, FAP, GD2, Glypican 3, Her 2, Mesothelin, and NKG2D, among others (134). However, current therapies face limitations, including off-target effects, fratricide, and challenges in identifying suitable TAA, especially in T-cell-derived cancers. These issues can lead to the destruction of healthy cells and diminished efficacy. CRISPR/Cas9 technology offers a solution by enabling precise gene editing to disrupt the expression of problematic antigens, such as CD3, CD5, and CD7, in CAR-T cells. This approach enhances antitumor efficacy while reducing fratricide and off-target effects, paving the way for more effective and safer CAR-T cell therapies.

Currently, only a limited number of clinical trials are employing CRISPR/Cas9 technology in CAR-T cells. For instance, NCT04037566 represents a pioneering trial evaluating CD19 CAR-T cells with edited endogenous HPK1 in patients with relapsed/refractory leukemia or lymphoma. Another trial, NCT04637763, is a phase I study investigating the efficacy and safety of CRISPR-edited allogeneic CD19 CAR-T cells in patients with relapsed/refractory B cell non-Hodgkin lymphoma. Stadtmauer et al. recently reported on a phase 1 clinical trial focusing on the safety and feasibility of CRISPR-Cas9 gene editing in three patients with advanced cancer. In this trial, T lymphocytes were extracted from patients and genetically modified using CRISPR-Cas9 to disrupt three genes (TRAC, TRBC, and PDCD1) to enhance antitumor immunity. Additionally, a cancer-targeting transgene, NY-ESO-1, was introduced to specifically target tumors. The engineered cells were administered to patients and demonstrated good tolerance, with sustained engraftment observed throughout the study period. These promising findings lay the groundwork for future trials exploring CRISPR-engineered cancer immunotherapies (135). Finally, NCT03545815 represents a phase I clinical trial employing CRISPR/Cas9 to disable PD-1 and TCR in CAR-T cells before administering them to patients with mesothelin-positive multiple solid tumors. Research has demonstrated that CRISPR-Cas9 can effectively disrupt up to five genes simultaneously in mouse embryonic stem cells with high efficiency (136). In another study, protocols were developed to efficiently generate CAR-T cells with edits in two genes (TRAC and B2M) or three genes (TRAC, B2M, and PD-1), evaluating their antitumor activities *in vitro*. Results indicated that CRISPR-Cas9-mediated multiple gene editing is readily applicable to CAR-T cells (137). However, CRISPR-edited CAR-T cells are still in early stages, and further preclinical studies, potentially conducted under Good Laboratory Practice (GLP), are necessary to pave the way for clinical trials.

The translation of CRISPR/Cas9 technology beyond CAR-T cells into clinical applications faces significant challenges that currently hinder its successful therapeutic implementation (Additional details are given in section 4). These challenges include, but are not limited to, several key issues. One major obstacle is the occurrence of off-target modifications, where the sgRNA can sometimes match with regions similar to the target sequence, leading Cas9 to cleave unintended off-target sites. Efforts to enhance the specificity of CRISPR/Cas9 have been pursued through improved gRNA design, the development of more efficient delivery vehicles, and the creation of novel Cas9 nucleases (109, 138, 139). Notably, newly designed variants like xCas9 and HypaCas9 appear to offer improved precision without compromising target activity (138, 140). Another concern associated with the use of CRISPR/Cas9 is its potential to introduce unintended deletions and complex genomic rearrangements into edited cells, which could pose irrecoverable genotoxicity risks in clinical applications (141). Addressing this challenge could involve strategies such as conducting whole-genome sequence analysis, employing in silico off-target prediction tools, assessing genotoxicity risks, and implementing long-term patient follow-up protocols (142, 143).

Additionally, the immunogenicity of the Cas9 protein presents another challenge that impedes the clinical adoption of the CRISPR/Cas9 system. Some individuals develop specific antibodies against the Cas9 protein, leading to T cell immune memory formation upon subsequent encounters. This immune response against Cas9 can diminish the editing efficiency and potentially lead to adverse effects. Moreover, CRISPR/Cas9 requires a specific PAM sequence (NGG) to exert its genome editing capability. The conventional Cas9 protein recognizes only a limited set of PAM sequences, restricting its versatility. However, the development of xCas9, an advanced variant, expands the range of PAM sequences recognized, thereby broadening the potential applications of the CRISPR/Cas9 system (138). Despite these advancements, the efficiency of HDR pathways for genomic insertion occasionally remains low (144). Strategies to address this issue include using single-stranded DNA templates instead of double-stranded DNA, inhibiting the NHEJ pathway, and employing advanced delivery methods like nucleofection (145, 146). Advancements in CAR-T cell design and the precision of CRISPR/Cas9 gene editing have paved the way for more effective and targeted therapies (**Table 3**).

**Table 3:** The integration of CAR-T and CRISPR/Cas9 in clinical settings.

Aspect	Description	Outcomes	Challenges	Ref
***Advancements in CAR-T Design***	Incorporation of costimulatory molecules, cytokines, and suicide genes to enhance efficacy and safety. Expanded tumor target repertoire.	Enhanced efficacy and safety in treating hematologic malignancies.	N/A	(132–134)
***Clinical Trials of CRISPR in CAR-T***	Trials like NCT04037566 and NCT04637763 are pioneering studies evaluating CRISPR-edited CAR-T cells. Stadtmauer et al.’s trial on CRISPR-Cas9 gene editing in advanced cancer patients.	Demonstrated feasibility and safety, sustained engraftment, and good tolerance.	Early stages of clinical trials; further studies needed for validation.	(135–137)
***Off-target Modifications***	Occurrence of unintended off-target modifications where sgRNA matches regions similar to the target sequence. Efforts to enhance specificity through improved gRNA design and novel Cas9 nucleases like xCas9 and HypaCas9.	Improved precision with xCas9 and HypaCas9 without compromising target activity.	Persistent risk of off-target effects; need for better prediction and assessment tools.	(109, 138–140)
***Genotoxicity Risks***	Potential for unintended deletions and complex genomic rearrangements posing genotoxicity risks. Strategies involve whole-genome sequence analysis, in silico off-target prediction, and long-term patient follow-up.	Genotoxicity risks need thorough assessment before clinical application	High genotoxicity risks require robust assessment and monitoring strategies.	(141–143)
***Immunogenicity and HDR Efficiency***	Cas9 protein immunogenicity leading to T cell immune memory formation. Limited PAM sequence recognition by conventional Cas9. xCas9 developed to recognize a broader range of PAM sequences. Efficiency of HDR pathways for genomic insertion is occasionally low.	Broadened potential applications with xCas9. Improved HDR efficiency with single-stranded DNA templates, NHEJ pathway inhibition, and advanced delivery methods.	Immunogenicity of Cas9 protein; limited HDR efficiency; strategies needed to overcome these challenges.	(99, 144–146)

Ref, References. “N/A” stands for “not available”.

Merging CRISPR/Cas9 with other emerging technologies, like mRNA vaccines and adoptive cell therapy, has the potential to dramatically impact cancer treatment. For instance, this could involve editing immune cells so they recognize and destroy cancerous cells, while mRNA vaccines prime the immune system against specific tumor-expressed antigens. Synergy in this direction may lead to more personalized, effective cancer therapies. Key findings suggest that CRISPR/Cas9 can greatly enhance the specificity and efficacy of CAR-T cells by deleting inhibitory genes or introducing new receptors that boost their function. However, challenges include ensuring the safety and precision of gene editing, preventing off-target effects, and optimizing delivery methods. Future integration of CAR-T cells with CRISPR/Cas9 may revolutionize cancer treatment by creating more potent, specific, and durable therapies. As these technologies advance, we may see next-generation CAR-T cells that are more effective against a broader range of cancers, safer, and tailored to individual patients. The future of cancer treatment may involve combining CRISPR-enhanced CAR-T cells, mRNA vaccines, and other innovative approaches to pave the way for breakthroughs in fighting cancer.

## 6 Personalized cancer therapies

The future of individualized therapy using combined approaches of CAR-T and CRISPR/Cas9 holds tremendous potential to revolutionize the treatment of cancer and genetic diseases. CAR-T therapy, which involves engineering a patient’s T-cells to target cancer cells, has shown remarkable success in treating certain blood cancers. Meanwhile, CRISPR/Cas9 has transformed genetic research by allowing precise modifications to DNA, making it a powerful tool for correcting genetic defects. By merging these two cutting-edge technologies, we can create more effective and personalized treatments tailored to individual patients’ genetic profiles. One of the most promising prospects of combining CAR-T and CRISPR/Cas9 is the enhancement of CAR-T cell therapy. CRISPR can be used to edit genes within T-cells to improve their ability to target and kill cancer cells. For example, CRISPR can knock out genes that inhibit T-cell activity or add genes that enhance their persistence and efficacy in the tumor microenvironment (147). Immune checkpoints, such as PD-1 and CTLA-4, are critical regulatory molecules in the immune system that prevent autoimmune reactions by dampening immune cell activity. However, this mechanism also allows cancer cells to evade immune responses. Immune checkpoint inhibitors work by blocking the interaction between these checkpoints and their ligands, thereby restoring T cell function. To counteract immune checkpoint inhibition in CAR-T cells, researchers have explored the combination of PD-1 inhibitors with CAR-T cell therapy. For instance, a research team engineered Mesothelin-CAR-T cells to treat pleural mesothelioma in mice and administered them alongside a PD-1 inhibitor. This combined approach effectively prolonged CAR-T cell activity, slowed tumor progression, and significantly extended median survival time (148). The feasibility of this combination therapy was further validated in a clinical trial, offering a promising strategy for enhancing cancer treatment outcomes (149). This synergy could potentially expand the success of CAR-T therapy beyond blood cancers to solid tumors, which have been challenging to treat with current CAR-T strategies. Moreover, the combined approach allows for the development of “off-the-shelf” CAR-T cells, which are derived from healthy donors rather than the patient. CRISPR/Cas9 can be used to edit these donor cells to prevent immune rejection and enhance their cancer-fighting properties (150).

This advancement would make CAR-T therapy more accessible, reducing the time and cost associated with generating personalized treatments from the patient’s own cells (151). Clinical applications face limitations due to the high cost of CAR-T cell therapy, which is mainly driven by complex manufacturing processes and expensive raw materials (14). Cost reductions can be achieved by improving the efficiency of production and using strict quality control systems to ensure consistency and reduce waste. A typical production cycle for CAR-T cells takes about two weeks, but new platforms are emerging that significantly reduce this time. For example, the Novartis T-Charge platform reduces *in vitro* culture time and increases T-cell proliferation (152). Dickinson et al. (153) developed CD19-CAR-T YTB323 autoimmune cell therapy using this platform in just two days. This is different from tisagenlecleucel, as YTB323 maintains T cell regulation and improves *in vivo* expansion and anti-tumor efficacy at low doses. Similarly, Grasel Biotechnologies’ FasTCAR platform has shortened the production time for CAR-T to one day. Clinical trials to evaluate the efficacy and safety of this process (NCT04638270, NCT05840107, and NCT04935580) are currently underway. In addition, CRISPR/Cas9 technology will accelerate more precise gene editing, allowing for rapid changes within a short period of time.

CRISPR can precisely correct genetic mutations in stem cells, which can then be differentiated into T-cells for CAR-T therapy. This approach not only targets cancer but also treats underlying genetic conditions, offering a dual therapeutic benefit. For example, patients with genetic immunodeficiencies could receive gene-corrected, cancer-targeting T-cells, addressing both their genetic disorder and cancer simultaneously (154). Despite these exciting prospects, challenges remain in ensuring the safety, efficacy, and ethical deployment of these therapies. Potential off-target effects of CRISPR and the long-term impacts of genetic modifications need thorough investigation. Regulatory frameworks must evolve to keep pace with technological advancements, ensuring that therapies are safe and ethically sound. Nonetheless, the combined use of CAR-T and CRISPR/Cas9 represents a promising frontier in personalized medicine, offering hope for more effective, targeted, and accessible treatments for cancer and genetic diseases.

## 7 Future directions

Editing CAR-T cells using CRISPR/Cas9 marks a pivotal advancement in the field of immunotherapy, addressing several critical challenges such as mitigating allogeneic reactions, overcoming tonic signaling and exhaustion within the tumor microenvironment (TME), and reducing potential toxicity. The scalability of CRISPR/Cas9 enables large-scale genetic screens, allowing researchers to efficiently and precisely investigate thousands of genes in T cells. This has led to groundbreaking preclinical studies that demonstrate the potential of CRISPR-edited CAR-T cells to significantly improve cancer treatment outcomes. As these technologies evolve, their integration into CAR-T cell therapy could revolutionize the approach to treating various forms of cancer, including those that have been resistant to conventional therapies.

Despite these promising advancements, there are still significant challenges that need to be addressed to fully realize the potential of CRISPR/Cas9 in CAR-T cell therapy. One of the primary concerns is the risk of off-target effects, where unintended genetic modifications could lead to adverse outcomes. Future research should focus on improving the precision of CRISPR/Cas9 through advancements in gRNA design, the development of novel Cas9 variants like xCas9 and HypaCas9, and the implementation of robust off-target detection methods. Additionally, optimizing the delivery systems for CRISPR/Cas9 components is crucial to ensure that the gene-editing machinery is accurately and efficiently introduced into target cells, minimizing the risk of unintended consequences.

Translating CRISPR/Cas9-engineered CAR-T cells into clinical practice also presents a set of challenges that must be carefully navigated. The transition from laboratory research to clinical application involves overcoming regulatory hurdles, designing clinical trials that accurately assess the safety and efficacy of these therapies, and scaling up production to meet the demands of a broader patient population. Establishing standardized manufacturing protocols and strict quality control measures will be essential to ensure the consistency and safety of CRISPR-edited CAR-T cells as they move toward widespread clinical use.

Looking forward, the future of CAR-T and CRISPR/Cas9 therapies could be further enhanced by integrating emerging technologies such as synthetic biology and artificial intelligence. Synthetic biology offers the potential to create programmable T cells that can be fine-tuned for specific therapeutic purposes, while artificial intelligence could be used to optimize gene-editing strategies and predict patient responses. These innovations, combined with ongoing advancements in CRISPR/Cas9 technology, could lead to even more personalized and effective cancer treatments, ultimately transforming the landscape of cancer care and offering new hope for patients facing some of the most challenging and refractory cancers.

## 8 Conclusion

The integration of CAR-T cell therapy with CRISPR/Cas9 technology has revolutionized cancer treatment by enhancing efficacy and personalization. CAR-T has shown success in treating hematologic cancers, but CRISPR/Cas9 offers precision and versatility to address limitations. By engineering CAR-T cells, researchers can enhance persistence, reduce off-target effects, and even knockout immune checkpoint inhibitors. However, challenges remain, such as off-target effects, optimizing gene-editing precision, and developing robust clinical protocols. Scaling up production, ensuring patient safety, and navigating regulatory landscapes are crucial steps for translating these therapies from preclinical success to widespread clinical use. Collaboration across academic, clinical, and industry sectors is essential to overcome barriers and accelerate the development of transformative therapies. The potential of CRISPR-edited CAR-T cells to revolutionize cancer treatment remains immense.

## References

[1] Jeschke J. Advances in cancer detection and diagnosis: from liquid biopsies and molecular biomarkers to opportunistic intratumoral bacteria. Curr Opin Oncol. (2023) 35:114. doi: 10.1097/CCO.0000000000000930 36721895

[2] Hofmarcher T Lindgren P Wilking N Jönsson B. The cost of cancer in Europe 2018. Eur J Cancer. (2020) 129:41–9. doi: 10.1016/j.ejca.2020.01.011 32120274

[3] Spokeviciute B Kholia S Brizzi MF. Chimeric antigen receptor (CAR) T-cell therapy: harnessing extracellular vesicles for enhanced efficacy. Pharmacol Res. (2024) 13:107352. doi: 10.1016/j.phrs.2024.107352 39147005

[4] Li X Li W Xu L Song Y. Chimeric antigen receptor-immune cells against solid tumors: Structures, mechanisms, recent advances, and future developments. Chin Med J. (2024) 137:1285–302. doi: 10.1097/CM9.0000000000002818 PMC11191032 37640679

[5] Pule MA Savoldo B Myers GD Rossig C Russell HV Dotti G. Virus-specific T cells engineered to coexpress tumor-specific receptors: persistence and antitumor activity in individuals with neuroblastoma. Nat Med. (2008) 14:1264. doi: 10.1038/nm.1882 18978797 PMC2749734

[6] Shademan B Karamad V Nourazarian A Avcı CB. CAR T cells: cancer cell surface receptors are the target for cancer therapy. Advanced Pharm bulletin. (2022) 12:476. doi: 10.34172/apb.2022.051 PMC9348524 35935042

[7] Yan W Liu Z Liu J Xia Y Hu K Yu J. Application of chimeric antigen receptor T cells in the treatment of hematological Malignancies. BioMed Res Int. (2020) 2020:1–9. doi: 10.1155/2020/4241864 PMC7547336 33062678

[8] Goldsmith SR Ghobadi A DiPersio JF. Hematopoeitic cell transplantation and CAR T-cell therapy: complements or competitors? Front Oncol. (2020) 10:2904. doi: 10.3389/fonc.2020.608916 PMC7783412 33415078

[9] Yang X Wang GX Zhou JF. CAR T cell therapy for hematological Malignancies. Curr Med Sci. (2019) 39:874–82. doi: 10.1007/s11596-019-2118-z 31845217

[10] Tao R Han X Bai X Yu J Ma Y Chen W. Revolutionizing cancer treatment: enhancing CAR-T cell therapy with CRISPR/Cas9 gene editing technology. Front Immunol. (2024) 15:1354825. doi: 10.3389/fimmu.2024.1354825 38449862 PMC10914996

[11] Fan J Adams A Sieg N Heger JM Gödel P Kutsch N. Potential synergy between radiotherapy and CAR T-cells-a multicentric analysis of the role of radiotherapy in the combination of CAR T cell therapy. Radiotherapy Oncol. (2023) 183:109580. doi: 10.1016/j.radonc.2023.109580 36842663

[12] Wang AX Ong XJ D’Souza C Neeson PJ Zhu JJ. Combining chemotherapy with CAR-T cell therapy in treating solid tumors. Front Immunol. (2023) 14:1140541. doi: 10.3389/fimmu.2023.1140541 36949946 PMC10026332

[13] Wang A Lv T Song Y. Tandem CAR-T cells targeting MUC1 and PSCA combined with anti-PD-1 antibody exhibit potent preclinical activity against non-small cell lung cancer. Cell Immunol. (2023) 391:104760. doi: 10.1016/j.cellimm.2023.104760 37660477

[14] Miao L Zhang Z Ren Z Tang F Li Y. Obstacles and coping strategies of CAR-T cell immunotherapy in solid tumors. Front Immunol. (2021) 12:687822. doi: 10.3389/fimmu.2021.687822 34093592 PMC8170155

[15] Shademan B Nourazarian A Hajazimian S Isazadeh A Biray Avci C Oskouee MA. CRISPR technology in gene-editing-based detection and treatment of SARS-CoV-2. Front Mol Biosciences. (2022) 8:772788. doi: 10.3389/fmolb.2021.772788 PMC8787289 35087864

[16] Cong L Ran FA Cox D Lin S Barretto R Habib N. Multiplex genome engineering using CRISPR/Cas systems. Science. (2013) 339:819–23. doi: 10.1126/science.1231143 PMC3795411 23287718

[17] Ghaffari S Khalili N Rezaei N. CRISPR/Cas9 revitalizes adoptive T-cell therapy for cancer immunotherapy. J Exp Clin Cancer Res. (2021) 40:269. doi: 10.1186/s13046-021-02076-5 34446084 PMC8390258

[18] Lana MG Strauss BE. Production of lentivirus for the establishment of CAR-T cells. Methods Mol Biol. (2020) 2086:61–7. doi: 10.1007/978-1-0716-0146-4_4 31707667

[19] Foster JB Choudhari N Perazzelli J Storm J Hofmann TJ Jain P. Purification of mRNA encoding chimeric antigen receptor is critical for generation of a robust T-cell response. Hum Gene Ther. (2019) 30:168–78. doi: 10.1089/hum.2018.145 PMC6383579 30024272

[20] Safarzadeh Kozani P Safarzadeh Kozani P Rahbarizadeh F. Novel antigens of CAR T cell therapy: new roads; old destination. Transl Oncol. (2021) 14:101079. doi: 10.1016/j.tranon.2021.101079 33862524 PMC8065293

[21] Safarzadeh Kozani P Safarzadeh Kozani P O'Connor RS. In like a lamb; out like a lion: marching CAR T cells toward enhanced efficacy in B-ALL. Mol Cancer Ther. (2021) 20:1223–33. doi: 10.1158/1535-7163.MCT-20-1089 PMC8285067 33903140

[22] Hashem Boroojerdi M Rahbarizadeh F Safarzadeh Kozani P Kamali E Safarzadeh KP. Strategies for having a more effective and less toxic CAR T-cell therapy for acute lymphoblastic leukemia. Med Oncol. (2020) 37:100. doi: 10.1007/s12032-020-01416-3 33047234 PMC7549730

[23] Safarzadeh Kozani P Safarzadeh Kozani P Rahbarizadeh F. CAR-T cell therapy in T-cell Malignancies: Is success a low-hanging fruit? Stem Cell Res Ther. (2021) 12:527. doi: 10.1186/s13287-021-02595-0 34620233 PMC8499460

[24] Safarzadeh Kozani P Safarzadeh Kozani P Rahbarizadeh F Khoshtinat NS. Strategies for Dodging the obstacles in CAR T cell therapy. Front Oncol. (2021) 11:627549. doi: 10.3389/fonc.2021.627549 33869011 PMC8047470

[25] Maude SL Laetsch TW Buechner J Rives S Boyer M Bittencourt H. Tisagenlecleucel in children and young adults with B-cell lymphoblastic leukemia. N Engl J Med. (2018) 378:439–48. doi: 10.1056/NEJMoa1709866 PMC5996391 29385370

[26] Maude SL Frey N Shaw PA Aplenc R Barrett DM Bunin NJ. Chimeric antigen receptor T cells for sustained remissions in leukemia. N Engl J Med. (2014) 371:1507–17. doi: 10.1056/NEJMoa1407222 PMC4267531 25317870

[27] Safarzadeh Kozani P Safarzadeh Kozani P Ahmadi Najafabadi M Yousefi F Mirarefin SM Rahbarizadeh F. Recent advances in solid tumor CAR-T cell therapy: driving tumor cells from hero to zero? Front Immunol. (2022) 13:795164. doi: 10.3389/fimmu.2022.795164 35634281 PMC9130586

[28] Holstein SA Lunning MA. CAR T-cell therapy in hematologic Malignancies: a voyage in progress. Clin Pharmacol Ther. (2020) 107:112–22. doi: 10.1002/cpt.v107.1 31622496

[29] Zhang X Zhu L Zhang H Chen S Xiao Y. CAR-T cell therapy in hematological Malignancies: current opportunities and challenges. Front Immunol. (2022) 13:927153. doi: 10.3389/fimmu.2022.927153 35757715 PMC9226391

[30] Pan J Yang JF Deng BP Zhao XJ Zhang X Lin YH. High efficacy and safety of low-dose CD19-directed CAR-T cell therapy in 51 refractory or relapsed B acute lymphoblastic leukemia patients. Leukemia. (2017) 31:2587–93. doi: 10.1038/leu.2017.145 28490811

[31] Sang W Shi M Yang J Cao J Xu L Yan D. Phase II trial of co-administration of CD19-and CD20-targeted chimeric antigen receptor T cells for relapsed and refractory diffuse large B cell lymphoma. Cancer Med. (2020) 9:5827–38. doi: 10.1002/cam4.v9.16 PMC7433814 32608579

[32] Olejniczak SH Stewart CC Donohue K Czuczman MS. A quantitative exploration of surface antigen expression in common B-cell Malignancies using flow cytometry. Immunol investigations. (2006) 35:93–114. doi: 10.1080/08820130500496878 16531332

[33] Haso W Lee DW Shah NN Stetler-Stevenson M Yuan CM Pastan IH. Anti-CD22–chimeric antigen receptors targeting B-cell precursor acute lymphoblastic leukemia. Blood J Am Soc Hematology. (2013) 121:1165–74. doi: 10.1182/blood-2012-06-438002 PMC3575759 23243285

[34] Pan J Niu Q Deng B Liu S Wu T Gao Z. CD22 CAR T-cell therapy in refractory or relapsed B acute lymphoblastic leukemia. Leukemia. (2019) 33:2854–66. doi: 10.1038/s41375-019-0488-7 31110217

[35] Fry TJ Shah NN Orentas RJ Stetler-Stevenson M Yuan CM Ramakrishna S. CD22-targeted CAR T cells induce remission in B-ALL that is naive or resistant to CD19-targeted CAR immunotherapy. Nat Med. (2018) 24:20–8. doi: 10.1038/nm.4441 PMC5774642 29155426

[36] Zhu H Deng H Mu J Lyu C Jiang Y Deng Q. Anti-CD22 CAR-T cell therapy as a salvage treatment in B cell Malignancies refractory or relapsed after anti-CD19 CAR-T therapy. OncoTargets Ther. (2021) 2:4023–37. doi: 10.2147/OTT.S312904 PMC8259947 34239307

[37] Tan Y Cai H Li C Deng B Song W Ling Z. A novel full-human CD22-CAR T cell therapy with potent activity against CD22low B-ALL. Blood Cancer J. (2021) 11:71. doi: 10.1038/s41408-021-00465-9 33839735 PMC8036232

[38] Qasim W Zhan H Samarasinghe S Adams S Amrolia P Stafford S. S. Molecular remission of infant B-ALL after infusion of universal TALEN gene-edited CAR T cells. Sci Trans Med. (2017) 9:eaaj2013. doi: 10.1126/scitranslmed.aaj2013 28123068

[39] Tashiro H. CAR T-cell therapy for T cell Malignancies: challenges and recent advances. Japanese J Clin Hematology. (2024) 65:644–51. doi: 10.11406/rinketsu.65.644 39098015

[40] Li S Wang X Yuan Z Liu L Luo L Li Y. Eradication of T-ALL cells by CD7-targeted universal CAR-T cells and initial test of ruxolitinib-based CRS management. Clin Cancer Res. (2021) 27:1242–6. doi: 10.1158/1078-0432.CCR-20-1271 33234511

[41] Pan J Tan Y Wang G Deng B Ling Z Song W. Donor-derived CD7 chimeric antigen receptor T cells for T-cell acute lymphoblastic leukemia: first-in-human, phase I trial. J Clin Oncol. (2021) 39:3340–51. doi: 10.1200/JCO.21.00389 34324392

[42] Xie L Ma L Liu S Chang L Wen F. Chimeric antigen receptor T cells targeting CD7 in a child with high-risk T-cell acute lymphoblastic leukemia. Int Immunopharmacology. (2021) 96:107731. doi: 10.1016/j.intimp.2021.107731 33965880

[43] Wada M Zhang H Fang L Feng J Tse CO Zhang W. Characterization of an anti-CD5 directed CAR T-cell against T-cell Malignancies. Stem Cell Rev Rep. (2020) 16:369–84. doi: 10.1007/s12015-019-09937-9 32008159

[44] Feng J Xu H Cinquina A Wu Z Chen Q Zhang P. Treatment of aggressive T cell lymphoblastic lymphoma/leukemia using anti-CD5 CAR T cells. Stem Cell Rev Rep. (2021) 17:652–61. doi: 10.1007/s12015-020-10092-9 PMC8036178 33410096

[45] Pinz K Liu H Golightly M Jares A Lan F Zieve GW. Preclinical targeting of human T-cell Malignancies using CD4-specific chimeric antigen receptor (CAR)-engineered T cells. Leukemia. (2016) 30:701–7. doi: 10.1038/leu.2015.311 26526988

[46] Alcantara M Tesio M June CH Houot R. CAR T-cells for T-cell Malignancies: challenges in distinguishing between therapeutic, normal, and neoplastic T-cells. Leukemia. (2018) 32:2307–15. doi: 10.1038/s41375-018-0285-8 PMC7433349 30315238

[47] Maciocia PM Wawrzyniecka PA Maciocia NC Burley A Karpanasamy T Devereaux S. Anti-CCR9 chimeric antigen receptor T cells for T-cell acute lymphoblastic leukemia. Blood J Am Soc Hematology. (2022) 140:25–37. doi: 10.1182/blood.2021013648 35507686

[48] Mardiana S Gill S. CAR T cells for acute myeloid leukemia: state of the art and future directions. Front Oncol. (2020) 10:697. doi: 10.3389/fonc.2020.00697 32435621 PMC7218049

[49] Bakker AB van den Oudenrijn S Bakker AQ Feller N van Meijer M Bia JA. C-type lectin-like molecule-1: a novel myeloid cell surface marker associated with acute myeloid leukemia. Cancer Res. (2004) 64:8443–50. doi: 10.1158/0008-5472.CAN-04-1659 15548716

[50] Wang J Chen S Xiao W Li W Wang L Yang S. CAR-T cells targeting CLL-1 as an approach to treat acute myeloid leukemia. J Hematol Oncol. (2018) 11:1–3. doi: 10.1186/s13045-017-0553-5 29316944 PMC5761206

[51] Lin G Zhang Y Yu L Wu D. Cytotoxic effect of CLL−1 CAR−T cell immunotherapy with PD−1 silencing on relapsed/refractory acute myeloid leukemia. Mol Med Rep. (2021) 23:1–. doi: 10.3892/mmr.2021.11847 PMC7830996 33495835

[52] Xie G Ivica NA Jia B Li Y Dong H Liang Y. CAR-T cells targeting a nucleophosmin neoepitope exhibit potent specific activity in mouse models of acute myeloid leukaemia. Nat Biomed Engineering. (2021) 5:399–413. doi: 10.1038/s41551-020-00625-5 PMC8039062 33046866

[53] Sauer T Parikh K Sharma S Omer B Sedloev D Chen Q. CD70-specific CAR T cells have potent activity against acute myeloid leukemia without HSC toxicity. Blood J Am Soc Hematology. (2021) 138:318–30. doi: 10.1182/blood.2020008221 PMC8323977 34323938

[54] Riether C Pabst T Höpner S Bacher U Hinterbrandner M Banz Y. Targeting CD70 with cusatuzumab eliminates acute myeloid leukemia stem cells in patients treated with hypomethylating agents. Nat Med. (2020) 26:1459–67. doi: 10.1038/s41591-020-0910-8 32601337

[55] John S Chen H Deng M Gui X Wu G Chen W. A novel anti-LILRB4 CAR-T cell for the treatment of monocytic AML. Mol Ther. (2018) 26:2487–95. doi: 10.1016/j.ymthe.2018.08.001 PMC6171100 30131301

[56] Jetani H Navarro-Bailón A Maucher M Frenz S Verbruggen C Yeguas A. Siglec-6 is a novel target for CAR T-cell therapy in acute myeloid leukemia. Blood J Am Soc Hematology. (2021) 138:1830–42. doi: 10.1182/blood.2020009192 PMC9642786 34289026

[57] Kim MY Yu K-R Kenderian SS Ruella M Chen S Shin TH. Genetic Inactivation of CD33 in hematopoietic stem cells to enable CAR T cell immunotherapy for acute myeloid leukemia. Cell. (2018) 173:1439–1453.e19. doi: 10.1016/j.cell.2018.05.013 29856956 PMC6003425

[58] Ferreri CJ Bhutani M. Mechanisms and management of CAR T toxicity. Front Oncol. (2024) 14:1396490. doi: 10.3389/fonc.2024.1396490 38835382 PMC11148294

[59] Zhang Y Qin D Shou AC Liu Y Wang Y Zhou L. Exploring CAR-T cell therapy side effects: mechanisms and management strategies. J Clin Med. (2023) 12:6124. doi: 10.3390/jcm12196124 37834768 PMC10573998

[60] Yáñez L Sánchez-Escamilla M Perales MA. CAR T cell toxicity: current management and future directions. Hemasphere. (2019) 3:e186. doi: 10.1097/HS9.0000000000000186 31723825 PMC6746032

[61] Gust J Hay KA Hanafi L-A Li D Myerson D Gonzalez-Cuyar LF. Endothelial activation and blood-brain barrier disruption in neurotoxicity after adoptive immunotherapy with CD19 CAR-T cells. Cancer Discovery. (2017) 7:1404–19. doi: 10.1158/2159-8290.CD-17-0698 PMC5718945 29025771

[62] Parker KR Migliorini D Perkey E Yost KE Bhaduri A Bagga P. Single-cell analyses identify brain mural cells expressing CD19 as potential off-tumor targets for CAR-T immunotherapies. Cell. (2020) 183:126–142.e17. doi: 10.1016/j.cell.2020.08.022 32961131 PMC7640763

[63] Neelapu SS Tummala S Kebriaei P Wierda W Gutierrez C Locke FL. Chimeric antigen receptor T-cell therapy—Assessment and management of toxicities. Nat Rev Clin Oncol. (2018) 15:47–62. doi: 10.1038/nrclinonc.2017.148 28925994 PMC6733403

[64] Hunter BD Jacobson CA. CAR T-cell associated neurotoxicity: mechanisms, clinicopathologic correlates, and future directions. J Natl Cancer Inst. (2019) 111:646–54. doi: 10.1093/jnci/djz017 30753567

[65] Freyer CW Porter DL. Cytokine release syndrome and neurotoxicity following CAR T-cell therapy for hematologic Malignancies. J Allergy Clin Immunol. (2020) 146:940–8. doi: 10.1016/j.jaci.2020.07.025 32771558

[66] Sheth VS Gauthier J. Taming the beast: CRS and ICANS after CAR T-cell therapy for ALL. Bone Marrow Transplant. (2021) 56:552–66. doi: 10.1038/s41409-020-01134-4 PMC8592274 33230186

[67] Barbar T Jaffer Sathick I. Tumor lysis syndrome. Adv Chronic Kidney Dis. (2021) 28:438–446.e1. doi: 10.1053/j.ackd.2021.09.007 35190110

[68] Maus MV Haas AR Beatty GL Albelda SM Levine BL Liu X. T cells expressing chimeric antigen receptors can cause anaphylaxis in humans. Cancer Immunol Res. (2013) 1:26–31. doi: 10.1158/2326-6066.CIR-13-0006 24777247 PMC3888798

[69] Kochenderfer JN Dudley ME Carpenter RO Kassim SH Rose JJ Telford WG. Donor-derived CD19-targeted T cells cause regression of Malignancy persisting after allogeneic hematopoietic stem cell transplantation. Blood. (2013) 122:4129–39. doi: 10.1182/blood-2013-08-519413 PMC3862276 24055823

[70] Morgan RA Yang JC Kitano M Dudley ME Laurencot CM Rosenberg SA. Case report of a serious adverse event following the administration of T cells transduced with a chimeric antigen receptor recognizing ERBB2. Mol Ther. (2010) 18:843–51. doi: 10.1038/mt.2010.24 PMC2862534 20179677

[71] Thistlethwaite FC Gilham DE Guest RD Rothwell DG Pillai M Burt DJ. The clinical efficacy of first-generation carcinoembryonic antigen (CEACAM5)-specific CAR T cells is limited by poor persistence and transient pre-conditioning-dependent respiratory toxicity. Cancer Immunol Immunother. (2017) 66:1425–36. doi: 10.1007/s00262-017-2034-7 PMC5645435 28660319

[72] Ramos CA Savoldo B Torrano V Ballard B Zhang H Dakhova O. Clinical responses with T lymphocytes targeting Malignancy-associated κ light chains. J Clin Invest. (2016) 126:2588–96. doi: 10.1172/JCI86000 PMC4922690 27270177

[73] Ranganathan R. Chimeric antigen receptor T cells targeting the lambda light chain of human immunoglobulin as a viable target for B cell non-Hodgkin lymphoma. J Clin Oncol. (2018) 36:12079. doi: 10.1200/JCO.2018.36.15_suppl.12079

[74] Li H Zhao Y. Increasing the safety and efficacy of chimeric antigen receptor T cell therapy. Protein Cell. (2017) 8:573–89. doi: 10.1007/s13238-017-0411-9 PMC5546931 28434147

[75] Chu F Cao J Neelalpu SS. Versatile CAR T-cells for cancer immunotherapy. Contemp Oncol (Poznan Poland). (2018) 22:73–80. doi: 10.5114/wo.2018.73892 PMC5885071 29628798

[76] Kochenderfer JN Dudley ME Feldman SA Wilson WH Spaner DE Maric I. B-cell depletion and remissions of Malignancy along with cytokine-associated toxicity in a clinical trial of anti-CD19 chimeric-antigen-receptor-transduced T cells. Blood. (2012) 119:2709–20. doi: 10.1182/blood-2011-10-384388 PMC3327450 22160384

[77] Brentjens RJ Davila ML Riviere I Park J Wang X Cowell LG. CD19-targeted T cells rapidly induce molecular remissions in adults with chemotherapy-refractory acute lymphoblastic leukemia. Sci Transl Med. (2013) 5:177ra38. doi: 10.1126/scitranslmed.3005930 PMC3742551 23515080

[78] Finney OC Brakke HM Rawlings-Rhea S Hicks R Doolittle D Lopez M. CD19 CAR T cell product and disease attributes predict leukemia remission durability. J Clin Investig. (2019) 129:2123–32. doi: 10.1172/JCI125423 PMC6486329 30860496

[79] Davila ML Riviere I Wang X Bartido S Park J Curran K. Efficacy and toxicity management of 19-28z CAR T cell therapy in B cell acute lymphoblastic leukemia. Sci Transl Med. (2014) 6:224ra25. doi: 10.1126/scitranslmed.3008226 PMC4684949 24553386

[80] Maalej KM Merhi M Inchakalody VP Mestiri S Alam M Maccalli C. CAR-cell therapy in the era of solid tumor treatment: current challenges and emerging therapeutic advances. Mol Cancer. (2023) 22:20. doi: 10.1186/s12943-023-01723-z 36717905 PMC9885707

[81] Pan K Farrukh H Chittepu VC Xu H Pan CX Zhu Z. CAR race to cancer immunotherapy: from CAR T, CAR NK to CAR macrophage therapy. J Exp Clin Cancer Res. (2022) 41:119. doi: 10.1186/s13046-022-02327-z 35361234 PMC8969382

[82] Furtado RN. Gene editing: the risks and benefits of modifying human DNA. Rev Bioetica. (2019) 27:223–33. doi: 10.1590/1983-80422019272304

[83] Novellasdemunt L Foglizzo V Cuadrado L Antas P Kucharska A Encheva V. USP7 is a tumor-specific WNT activator for APC-mutated colorectal cancer by mediating β-catenin deubiquitination. Cell Rep. (2017) 21:612–27. doi: 10.1016/j.celrep.2017.09.072 PMC5656747 29045831

[84] Brunet E Jasin M. Induction of chromosomal translocations with CRISPR-Cas9 and other nucleases: understanding the repair mechanisms that give rise to translocations. Chromosome Translocation. (2018) 1044:15–25. 10.1007/978-981-13-0593-1_2 PMC6333474 29956288

[85] Costa JR Bejcek BE McGee JE Fogel AI Brimacombe KR Ketteler R. Genome editing using engineered nucleases and their use in genomic screening. In: Assay Guidance Manual (2017).

[86] Shademan B Masjedi S Karamad V Isazadeh A Sogutlu F Rad MH. CRISPR technology in cancer diagnosis and treatment: opportunities and challenges. Biochem Genet. (2022) 60:1446–70. doi: 10.1007/s10528-022-10193-9 35092559

[87] Gupta RM Musunuru K. Expanding the genetic editing tool kit: ZFNs, TALENs, and CRISPR-Cas9. J Clin Invest. (2014) 124:4154–61. doi: 10.1172/JCI72992 PMC4191047 25271723

[88] Sekine R Kawata T Muramoto T. CRISPR/Cas9 mediated targeting of multiple genes in Dictyostelium. Sci Rep. (2018) 8:8471. doi: 10.1038/s41598-018-26756-z 29855514 PMC5981456

[89] Zhan T Rindtorff N Betge J Ebert MP Boutros M. CRISPR/Cas9 for cancer research and therapy. In: Seminars in Cancer Biology, vol. 55. Academic Press (2019). p. 106–19. 10.1016/j.semcancer.2018.04.001 29673923

[90] Martinez-Lage M Torres-Ruiz R Puig-Serra P Moreno-Gaona P Martin MC Moya FJ. *In vivo* CRISPR/Cas9 targeting of fusion oncogenes for selective elimination of cancer cells. Nat Commun. (2020) 11:5060. doi: 10.1038/s41467-020-18875-x 33033246 PMC7544871

[91] Fellmann C Gowen BG Lin PC Doudna JA Corn JE. Cornerstones of CRISPR-Cas in drug discovery and therapy. Nat Rev Drug Discovery. (2017) 16:89–100. doi: 10.1038/nrd.2016.238 28008168 PMC5459481

[92] Meyers RM Bryan JG McFarland JM Weir BA Sizemore AE Xu H. Computational correction of copy number effect improves specificity of CRISPR-Cas9 essentiality screens in cancer cells. Nat Genet. (2017) 49:1779–84. doi: 10.1038/ng.3984 PMC5709193 29083409

[93] Behan FM Iorio F Picco G Gonçalves E Beaver CM Migliardi G. Prioritization of cancer therapeutic targets using CRISPR-Cas9 screens. Nature. (2019) 568:511–6. doi: 10.1038/s41586-019-1103-9 30971826

[94] Chen S Sanjana NE Zheng K Shalem O Lee K Shi X. Genome-wide CRISPR screen in a mouse model of tumor growth and metastasis. Cell. (2015) 160:1246–60. doi: 10.1016/j.cell.2015.02.038 PMC4380877 25748654

[95] Chow RD Guzman CD Wang G Schmidt F Youngblood MW Ye L. AAV-mediated direct *in vivo* CRISPR screen identifies functional suppressors in glioblastoma. Nat Neurosci. (2017) 20:1329–41. doi: 10.1038/nn.4620 PMC5614841 28805815

[96] Zhao Z Li C Tong F Deng J Huang G Sang Y. Review of applications of CRISPR-Cas9 gene-editing technology in cancer research. Biol Procedures Online. (2021) 23:1–3. doi: 10.1186/s12575-021-00151-x PMC8281662 34261433

[97] Asad AS Moreno Ayala MA Gottardo MF Zuccato C Nicola Candia AJ Zanetti FA. Viral gene therapy for breast cancer: progress and challenges. Expert Opin Biol Ther. (2017) 17:945–59. doi: 10.1080/14712598.2017.1338684 28604109

[98] Gaj T Epstein BE Schaffer DV. Genome engineering using adeno-associated virus: basic and clinical research applications. Mol Ther. (2016) 24:458–64. doi: 10.1038/mt.2015.151 PMC4786909 26373345

[99] Alves E Taifour S Dolcetti R Chee J Nowak AK Gaudieri S. Reprogramming the anti-tumor immune response via CRISPR genetic and epigenetic editing. Mol Therapy-Methods Clin Dev. (2021) 21:592–606. doi: 10.1016/j.omtm.2021.04.009 PMC8142043 34095343

[100] Qi LS Larson MH Gilbert LA Doudna JA Weissman JS Arkin AP. Repurposing CRISPR as an RNA-guided platform for sequence-specific control of gene expression. Cell. (2013) 152:1173–83. doi: 10.1016/j.cell.2013.02.022 PMC3664290 23452860

[101] Chavez A Scheiman J Vora S Pruitt BW Tuttle M PR Iyer E. Highly efficient Cas9-mediated transcriptional programming. Nat Methods. (2015) 12:326–8. doi: 10.1038/nmeth.3312 PMC4393883 25730490

[102] Ureña-Bailén G Lamsfus-Calle A Daniel-Moreno A Raju J Schlegel P Seitz C. CRISPR/Cas9 technology: towards a new generation of improved CAR-T cells for anticancer therapies. Briefings Funct Genomics. (2020) 19:191–200. doi: 10.1093/bfgp/elz039 31844895

[103] Mali P Esvelt KM Church GM. Cas9 as a versatile tool for engineering biology. Nat Methods. (2013) 10:957–63. doi: 10.1038/nmeth.2649 PMC4051438 24076990

[104] Teimourian S Abdollahzadeh R. Technology developments in biological tools for targeted genome surgery. Biotechnol Lett. (2015) 37:29–39. doi: 10.1007/s10529-014-1656-5 25257583

[105] Dötsch S Svec M Schober K Hammel M Wanisch A Gökmen F. Long-term persistence and functionality of adoptively transferred antigen-specific T cells with genetically ablated PD-1 expression. Proc Natl Acad Sci. (2023) 120:e2200626120. doi: 10.1073/pnas.2200626120 36853939 PMC10013756

[106] Agarwal S Aznar MA Rech AJ Good CR Kuramitsu S Da T. Deletion of the inhibitory co-receptor CTLA-4 enhances and invigorates chimeric antigen receptor T cells. Immunity. (2023) 56:2388–407. doi: 10.1016/j.immuni.2023.09.001 PMC10591801 37776850

[107] Eyquem J Mansilla-Soto J Giavridis T van der Stegen SJ Hamieh M Cunanan KM. Targeting a CAR to the TRAC locus with CRISPR/Cas9 enhances tumour rejection. Nature. (2017) 543:113. doi: 10.1038/nature21405 28225754 PMC5558614

[108] Fu Y Sander JD Reyon D Cascio VM Joung JK. Improving CRISPR-Cas nuclease specificity using truncated guide RNAs. Nat Biotechnol. (2014) 32:279. doi: 10.1038/nbt.2808 24463574 PMC3988262

[109] Kleinstiver BP Pattanayak V Prew MS Tsai SQ Nguyen NT Zheng Z. High-fidelity CRISPR–Cas9 nucleases with no detectable genome-wide off-target effects. Nature. (2016) 529:490–5. doi: 10.1038/nature16526 PMC4851738 26735016

[110] Yin H Song C-Q Suresh S Kwan SY Wu Q Walsh S. Partial DNA-guided Cas9 enables genome editing with reduced off-target activity. Nat Chem Biol. (2018) 14:311. doi: 10.1038/nchembio.2559 29377001 PMC5902734

[111] Slaymaker IM Gao L Zetsche B Scott DA Yan WX Zhang F. Rationally engineered Cas9 nucleases with improved specificity. Science. (2016) 351:84–8. doi: 10.1126/science.aad5227 PMC4714946 26628643

[112] Maruyama T Dougan SK Truttmann MC Bilate AM Ingram JR Ploegh HL. Increasing the efficiency of precise genome editing with CRISPR-Cas9 by inhibition of nonhomologous end joining. Nat Biotechnol. (2015) 33:538. doi: 10.1038/nbt.3190 25798939 PMC4618510

[113] Paulsen S Mandal BP Frock R Boyraz B Yadav R Upadhyayula S. Ectopic expression of RAD52 and dn53BP1 improves homology-directed repair during CRISPR–Cas9 genome editing. Nat BioMed Eng. (2017) 1:878–88. doi: 10.1038/s41551-017-0145-2 PMC6918705 31015609

[114] Song J Yang D Xu J Zhu T Chen YE Zhang J. RS-1 enhances CRISPR/Cas9- and TALEN-mediated knock-in efficiency. Nat Commun. (2016) 7:10548. doi: 10.1038/ncomms10548 26817820 PMC4738357

[115] Ren J Liu X Fang C Jiang S June CH Zhao Y. Multiplex genome editing to generate universal CAR T cells resistant to PD1 inhibition. Clin Cancer Res. (2017) 23:2255–66. doi: 10.1158/1078-0432.CCR-16-1300 PMC5413401 27815355

[116] Zhang Y Zhang X Cheng C Mu W Liu X Li N. CRISPR-Cas9 mediated LAG-3 disruption in CAR-T cells. Front Med. (2017) 11:554–62. doi: 10.1007/s11684-017-0543-6 28625015

[117] Guo X Jiang H Shi B Zhou M Zhang H Shi Z. Disruption of PD-1 enhanced the anti-tumor activity of chimeric antigen receptor T cells against hepatocellular carcinoma. Front Pharmacol. (2018) 9:1118. doi: 10.3389/fphar.2018.01118 30327605 PMC6174208

[118] Jung IY Kim YY Yu HS Lee M Kim S Lee J. CRISPR/Cas9-mediated knockout of DGK improves antitumor activities of human T cells. Cancer Res. (2018) 78:4692–703. doi: 10.1158/0008-5472.CAN-18-0030 29967261

[119] Cooper ML Choi J Staser K Ritchey JK Devenport JM Eckardt K. An “off-the-shelf” fratricide-resistant CAR-T for the treatment of T cell hematologic Malignancies. Leukemia. (2018) 32:1970–83. doi: 10.1038/s41375-018-0065-5 PMC6102094 29483708

[120] Ren J Zhang X Liu X Fang C Jiang S June CH. A versatile system for rapid multiplex genome-edited CAR T cell generation. Oncotarget. (2017) 8:17002. doi: 10.18632/oncotarget.15218 28199983 PMC5370017

[121] Jung I-Y Lee J. Unleashing the therapeutic potential of CAR-T cell therapy using gene-editing technologies. Mol Cells. (2018) 41:717–23. doi: 10.14348/molcells.2018.0242 PMC6125425 30110720

[122] Scherer LD Brenner MK Mamonkin M. Chimeric antigen receptors for T-cell Malignancies. Front Oncol. (2019) 9:126. doi: 10.3389/fonc.2019.00126 30891427 PMC6411696

[123] Cooper ML Choi J Staser K Ritchey JK Devenport JM Eckardt K. An "off-the-shelf" fratricide-resistant CAR-T for the treatment of T cell hematologic Malignancies. Leukemia. (2018) 32:1970–83. doi: 10.1038/s41375-018-0065-5 PMC6102094 29483708

[124] Fleischer LC Raikar SS Moot R Knight KA Doering CB Spencer HT. Engineering CD5-targeted chimeric antigen receptors and edited T cells for the treatment of T-cell leukemia. Blood. (2017) 130:1914. doi: 10.1182/blood.V130.Suppl_1.1914.1914

[125] Rasaiyaah J Georgiadis C Preece R Preece R Mock U Qasim W. TCRαβ/CD3 disruption enables CD3-specific antileukemic T cell immunotherapy. JCI Insight. (2018) 3:e99442. doi: 10.1172/jci.insight.99442 29997304 PMC6124532

[126] Galetto R Chion-Sotinel I Gouble A Smith J. Bypassing the constraint for chimeric antigen receptor (CAR) development in T-cells expressing the targeted antigen: improvement of anti-CS1 CAR activity in allogenic TCRa/CS1 double knockout T-cells for the treatment of multiple myeloma (MM). Blood. (2015) 126:116. doi: 10.1182/blood.V126.23.116.116

[127] Antony JS Haque AKMA Lamsfus-Calle A Daniel-Moreno A Mezger M Kormann MS. CRISPR/Cas9 system: a promising technology for the treatment of inherited and neoplastic hematological diseases. Adv Cell Gene Ther. (2018) 1:e10. doi: 10.1002/acg2.v1.1

[128] Cavazzana-Calvo M Payen E Negre O Wang G Hehir K Fusil F. Transfusion independence and HMGA2 activation after gene therapy of human β-thalassaemia. Nature. (2010) 467:318–22. doi: 10.1038/nature09328 PMC3355472 20844535

[129] Roth TL Puig-Saus C Yu R Shifrut E Carnevale J Li PJ. Reprogramming human T cell function and specificity with non-viral genome targeting. Nature. (2018) 559:405–9. doi: 10.1038/s41586-018-0326-5 PMC6239417 29995861

[130] Banasik MB McCray PB Jr. Integrase-defective lentiviral vectors: progress and applications. Gene Ther. (2009) 17:150. doi: 10.1038/gt.2009.135 19847206

[131] Genovese P Schiroli G Escobar G Di Tomaso T Firrito C Calabria A. Targeted genome editing in human repopulating haematopoietic stem cells. Nature. (2014) 510:235. doi: 10.1038/nature13420 24870228 PMC4082311

[132] Sadelain M Brentjens R Rivière I. The basic principles of chimeric antigen receptor design. Cancer discovery. (2013) 3:388–98. doi: 10.1158/2159-8290.CD-12-0548 PMC3667586 23550147

[133] Wang X Rivière I. Clinical manufacturing of CAR T cells: foundation of a promising therapy. Mol Therapy-Oncolytics. (2016) 3:16015. doi: 10.1038/mto.2016.15 PMC4909095 27347557

[134] Gill S Maus MV Porter DL. Chimeric antigen receptor T cell therapy: 25 years in the making. Blood Rev. (2016) 30:157–67. doi: 10.1016/j.blre.2015.10.003 26574053

[135] Stadtmauer EA Fraietta JA Davis MM Cohen AD Weber KL Lancaster E. CRISPR-engineered T cells in patients with refractory cancer. Science. (2020) 367:eaba7365. doi: 10.1126/science.aba7365 32029687 PMC11249135

[136] Wang H Yang H Shivalila CS Dawlaty MM Cheng AW Zhang F. One-step generation of mice carrying mutations in multiple genes by CRISPR/Cas-mediated genome engineering. Cell. (2013) 153:910–8. doi: 10.1016/j.cell.2013.04.025 PMC3969854 23643243

[137] Liu X Zhang Y Cheng C Cheng AW Zhang X Li N. CRISPR-Cas9-mediated multiplex gene editing in CAR-T cells. Cell Res. (2017) 27:154–7. doi: 10.1038/cr.2016.142 PMC5223227 27910851

[138] Hu JH Miller SM Geurts MH Tang W Chen L Sun N. Evolved Cas9 variants with broad PAM compatibility and high DNA specificity. Nature. (2018) 556:57–63. doi: 10.1038/nature26155 29512652 PMC5951633

[139] Shen B Zhang W Zhang J Zhou J Wang J Chen L. Efficient genome modification by CRISPR-Cas9 nickase with minimal off-target effects. Nat Methods. (2014) 11:399–402. doi: 10.1038/nmeth.2857 24584192

[140] Chen JS Dagdas YS Kleinstiver BP Welch MM Sousa AA Harrington LB. Enhanced proofreading governs CRISPR–Cas9 targeting accuracy. Nature. (2017) 550:407–10. doi: 10.1038/nature24268 PMC5918688 28931002

[141] Shin HY Wang C Lee HK Yoo KH Zeng X Kuhns T. CRISPR/Cas9 targeting events cause complex deletions and insertions at 17 sites in the mouse genome. Nat Commun. (2017) 8:15464. doi: 10.1038/ncomms15464 28561021 PMC5460021

[142] Hsu PD Scott DA Weinstein JA Ran FA Konermann S Agarwala V. DNA targeting specificity of RNA-guided Cas9 nucleases. Nat Biotechnol. (2013) 31:827–32. doi: 10.1038/nbt.2647 PMC3969858 23873081

[143] Zhang XH Tee LY Wang XG Huang QS Yang SH. Off-target effects in CRISPR/Cas9-mediated genome engineering. Mol Therapy-Nucleic Acids. (2015) 4:e264. doi: 10.1038/mtna.2015.37 PMC4877446 26575098

[144] Komor AC Badran AH Liu DR. CRISPR-based technologies for the manipulation of eukaryotic genomes. Cell. (2017) 168:20–36. doi: 10.1016/j.cell.2016.10.044 27866654 PMC5235943

[145] Chu VT Weber T Wefers B Wurst W Sander S Rajewsky K. Increasing the efficiency of homology-directed repair for CRISPR-Cas9-induced precise gene editing in mammalian cells. Nat Biotechnol. (2015) 33:543–8. doi: 10.1038/nbt.3198 25803306

[146] Richardson CD Ray GJ DeWitt MA Curie GL Corn JE. Enhancing homology-directed genome editing by catalytically active and inactive CRISPR-Cas9 using asymmetric donor DNA. Nat Biotechnol. (2016) 34:339–44. doi: 10.1038/nbt.3481 26789497

[147] Wei W Chen ZN Wang K. CRISPR/Cas9: A powerful strategy to improve CAR-T cell persistence. Int J Mol Sci. (2023) 24:12317. doi: 10.3390/ijms241512317 37569693 PMC10418799

[148] Cherkassky L Morello A Villena-Vargas J Feng Y Dimitrov DS Jones DR. Human CAR T cells with cell-intrinsic PD-1 checkpoint blockade resist tumor-mediated inhibition. J Clin Invest. (2016) 126:3130–44. doi: 10.1172/JCI83092 PMC4966328 27454297

[149] Adusumilli PS Zauderer MG Rivière I Solomon SB Rusch VW O'Cearbhaill RE. A phase I trial of regional mesothelin-targeted CAR T-cell therapy in patients with Malignant pleural disease, in combination with the anti–PD-1 agent pembrolizumab. Cancer Discovery. (2021) 11:2748–63. doi: 10.1158/2159-8290.CD-21-0407 PMC8563385 34266984

[150] Aparicio C Acebal C González-Vallinas M. Current approaches to develop “off-the-shelf” chimeric antigen receptor (CAR)-T cells for cancer treatment: a systematic review. Exp Hematol Oncol. (2023) 12:73. doi: 10.1186/s40164-023-00435-w 37605218 PMC10440917

[151] Cutmore LC Marshall JF. Current perspectives on the use of off the shelf CAR-T/NK cells for the treatment of cancer. Cancers. (2021) 13:1926. doi: 10.3390/cancers13081926 33923528 PMC8074108

[152] Ghassemi S Durgin JS Nunez-Cruz S Patel J Leferovich J Pinzone M. Rapid manufacturing of non-activated potent CAR T cells. Nat Biomed Engineering. (2022) 6:118–28. doi: 10.1038/s41551-021-00842-6 PMC8860360 35190680

[153] Dickinson MJ Barba P Jäger U Shah NN Blaise D Briones J. A novel autologous CAR-T therapy, YTB323, with preserved T-cell stemness shows enhanced CAR T-cell efficacy in preclinical and early clinical development. Cancer Discovery. (2023) 13:1982–97. doi: 10.1158/2159-8290.CD-22-1276 PMC10481129 37249512

[154] Gao Q Dong X Xu Q Zhu L Wang F Hou Y. Therapeutic potential of CRISPR/Cas9 gene editing in engineered T-cell therapy. Cancer Med. (2019) 8:4254–64. doi: 10.1002/cam4.2257 PMC6675705 31199589

